# CRISPR/Cas9-based genetic correction for recessive dystrophic epidermolysis bullosa

**DOI:** 10.1038/npjregenmed.2016.14

**Published:** 2016-12-08

**Authors:** Beau R Webber, Mark J Osborn, Amber N McElroy, Kirk Twaroski, Cara-lin Lonetree, Anthony P DeFeo, Lily Xia, Cindy Eide, Christopher J Lees, Ron T McElmurry, Megan J Riddle, Chong Jai Kim, Dharmeshkumar D Patel, Bruce R Blazar, Jakub Tolar

**Affiliations:** 1 Department of Pediatrics, Division of Blood and Marrow Transplantation, University of Minnesota, Minneapolis, MN, USA; 2 Stem Cell Institute, University of Minnesota, Minneapolis, MN, USA; 3 Center for Genome Engineering, University of Minnesota, Minneapolis, MN, USA; 4 Asan-Minnesota Institute for Innovating Transplantation, Seoul, Republic of Korea

## Abstract

Recessive dystrophic epidermolysis bullosa (RDEB) is a severe disorder caused by mutations to the *COL7A1* gene that deactivate production of a structural protein essential for skin integrity. Haematopoietic cell transplantation can ameliorate some of the symptoms; however, significant side effects from the allogeneic transplant procedure can occur and unresponsive areas of blistering persist. Therefore, we employed genome editing in patient-derived cells to create an autologous platform for multilineage engineering of therapeutic cell types. The clustered regularly interspaced palindromic repeats (CRISPR)/Cas9 system facilitated correction of an RDEB-causing *COL7A1* mutation in primary fibroblasts that were then used to derive induced pluripotent stem cells (iPSCs). The resulting iPSCs were subsequently re-differentiated into keratinocytes, mesenchymal stem cells (MSCs) and haematopoietic progenitor cells using defined differentiation strategies. Gene-corrected keratinocytes exhibited characteristic epithelial morphology and expressed keratinocyte-specific genes and transcription factors. iPSC-derived MSCs exhibited a spindle morphology and expression of CD73, CD90 and CD105 with the ability to undergo adipogenic, chondrogenic and osteogenic differentiation *in vitro* in a manner indistinguishable from bone marrow-derived MSCs. Finally, we used a vascular induction strategy to generate potent definitive haematopoietic progenitors capable of multilineage differentiation in methylcellulose-based assays. In totality, we have shown that CRISPR/Cas9 is an adaptable gene-editing strategy that can be coupled with iPSC technology to produce multiple gene-corrected autologous cell types with therapeutic potential for RDEB.

## Introduction

Recessive dystrophic epidermolysis bullosa (RDEB) is a monogenic disorder resulting from mutations in the type VII collagen gene (*COL7A1*) on chromosome 3. The mutational profile can be heterogeneic in regards to position and can encompass homozygous or compound heterozygous alterations.^1^ The resultant loss of the functional type VII collagen protein (C7) at the dermal-epidermal junction compromises the integrity of the attachment of the epidermis to the dermis, resulting in severe blistering, fibrosis and a predisposition to squamous cell carcinoma. Non-cutaneous manifestations, including corneal and oesophageal lesions, further contribute to a pathogenic state leading to a multi-decade decrease in life expectancy.

Treatment for RDEB includes palliative bandaging of active wounds and pain management, as well as allogeneic and autologous cellular therapy. Palliation is non-curative, and cellular therapy can include localised injection of type VII collagen-expressing cells and/or systemic infusion of haematopoietic stem/progenitor cells (HSPCs) that repopulate the host with donor-derived cells.^2^ Keratinocytes and fibroblasts represent the major C7 producing cells of the skin; however, their poor *in vitro* proliferative and expansion properties as primary cells limit their therapeutic potential and impact. Mesenchymal stromal/stem cells (MSCs) have been used as a supportive therapy and possess wound migratory potential and the ability to actively participate in, as well as to orchestrate, healing.^3,4^ Similar to other primary cells, primary bone marrow-derived MSCs can senesce and lose their beneficial properties with *in vitro* expansion.

Towards mediating systemic effects, allogeneic haematopoietic cell transplant (HCT) has been employed. HCT has resulted in significant, but neither uniform nor complete, outcomes.^5^ For each modality, the use of allogeneic cells limits efficacy. Locally injected cells appear to persist transiently, likely due to immune clearance, necessitating repeated injections that is limiting in terms of the difficulty in long-term culture/maintenance, surface area able to be treated, and availability of allogeneic cells that can be obtained, archived and expanded for subsequent injections.^6^ HCT can result in graft-versus-host disease that can cause severe side effects, making the use of autologous cells highly desirous. To realise the potential of such an approach, we set out to determine whether an RDEB patient’s *COL7A1* gene defect could be restored to wild-type status in a population of cells that could be utilised as a template for sustainable multilineage progeny generation.

Two major platforms exist for facilitating gene correction: gene therapy and gene editing. Gene therapy for RDEB has centred primarily on lentiviral gene transfer of a copy of the *COL7A1* cDNA, expression of which is governed by exogenous regulatory elements.^7,8^ While this strategy meets the need for autologous cellular engineering, there are significant hurdles to this approach. The large size of the cDNA can negatively impact viral titres making manufacturing, production and efficient gene delivery rates suboptimal. Further, the integrating properties of vectors capable of long-term gene expression represent a risk for insertional mutagenic-derived adverse events. This fact is particularly relevant given that RDEB patients are at an increased risk for squamous cell carcinoma, thus potentially placing cutaneous cells in a pre-malignant state that are less tolerant to the genomic perturbation that accompanies integrating vectors.^9^ In addition, the artificial expression cassette components are not subject to the normal cellular gene regulatory environment. This point is highly relevant, as perturbation of ECM protein expression has been shown to impact the cellular microenvironment, and the long-term effect of supra-physiological *COL7A1* expression is unknown. These considerations make gene editing the preferred methodology for autologous cell precision correction *in situ* that mitigates genomic toxicity and maintains the endogenous cellular gene expression command and control system.

Gene targeting at translational efficiency requires site-specific reagents that cleave the DNA helix, and there are multiple candidates capable of accomplishing this. Zinc finger nucleases and transcription activator-like effector nucleases function as dimeric proteins that co-localise at a target site and mediate a double-stranded DNA break.^10–12^ Meganucleases are a monomeric protein specified for a unique sequence utilising a bacterial endonuclease as the engineering template.^13^ The clustered regularly interspaced palindromic repeats (CRISPR)/Cas9 reagent is a two-component system consisting of the Cas9 nuclease that conjugates with a small RNA transcript termed a guide RNA (gRNA). This complex interacts with a target sequence consisting of 15–20 nucleotides. Once a DNA break is generated, genome modification by homology-directed repair (HDR) can occur using an exogenous donor DNA species as the repair template.

Here we utilised the CRISPR/Cas9 system to facilitate gene repair in cells from an RDEB patient. Correction of *COL7A1* fibroblasts was achieved, and these fibroblasts were used as a renewable template for patient-specific induced pluripotent stem cell (iPSC) derivation. To capitalise on the ease of continuous propagation and broad differentiation potential of iPSCs, they were employed in a therapeutic engineering strategy to generate keratinocytes, MSCs and haematopoietic cells. This multilineage approach represents a strategy for broad therapeutic use in support of combinatorial systemic and localised interventions.^14^

## Results

**Figure 1:**
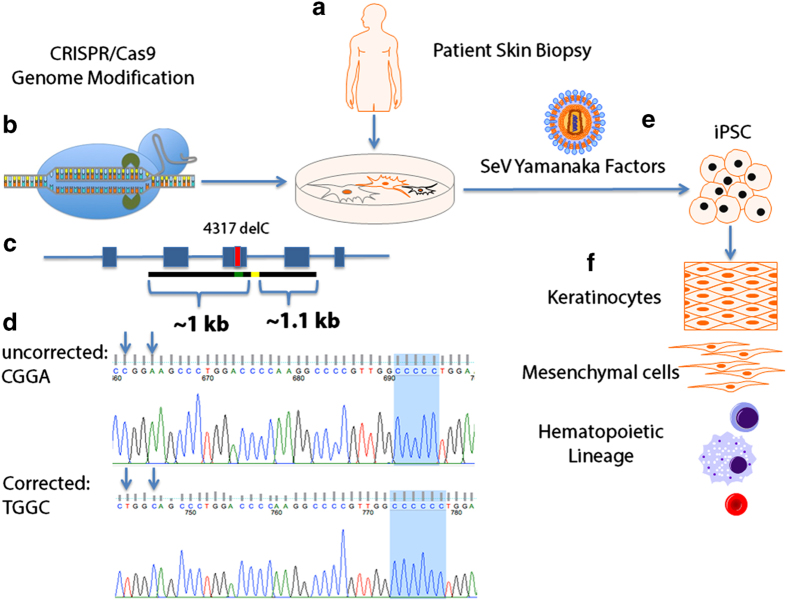
Experimental schema for gene correction and cellular engineering. (**a**) A punch biopsy was obtained for primary fibroblast cell derivation. (**b**) The CRISPR/Cas9 gene-editing platform was employed for 4317delC *COL7A1* gene correction. (**c**) *COL7A1* locus and gene repair template. The 4317delC mutation is indicated with a red box. The donor template was a plasmid containing ~1 kb of homology to the target sequence and flanked a floxed PGK puromycin selection cassette (yellow box). The cytosine to restore proper genotype and two silent polymorphisms were introduced into the donor arm and is indicated with a green box. (**d**) *COL7A1* locus correction. Sanger sequence of uncorrected cells before treatment showing a deletion of a single cytosine and unmodified base sequences (top). Subsequent CRISPR/Cas9 mediated repair by the donor resulted in restoration of the deleted cytosine (shaded in blue) and incorporation of engineered marker SNPs (blue arrows). (**e**) The corrected fibroblasts were reprogrammed to pluripotency using Sendai virus delivery of the reprogramming factors. (**f**) iPSCs served as a template for directed differentiation into keratinocytes, mesenchymal stem cells and haematopoietic progenitors.

The overall experimental schema is detailed in Figure 1 and is a strategy that employed patient-derived fibroblasts that were precisely modified with a CRISPR/Cas9 mutation-specific reagent. Cells were induced to pluripotency with Sendai viral, footprint-free reprogramming and differentiated into effector lineages.

### CRISPR/Cas9 gene correction

We generated three candidate gRNAs proximal to the 4317delC *COL7A1* gene mutation characterised by a single cytosine deletion (Supplementary Figures S1a and b) for testing with the *S. pyogenes* Cas9 that was delivered as DNA expression cassettes (Supplementary Figures S1c and d). Using the Surveyor assay,^15^ all three candidates resulted in cleavage products consistent with Cas9 activity and DNA repair by non-homologous end joining (Supplementary Figures S1e and f). Because fibroblasts are a major contributor of C7 in the dermis, we deployed CRISPR/Cas9 reagents for gene correction using a targeting strategy comprised of three plasmids: Cas9 nuclease or nickase, the C7 gRNA, and an exogenous donor repair template (Figure 1c and Supplementary Figure S2a). The Cas9 nuclease generates double-stranded DNA breaks while the nickase version, due to inactivation of the Cas9 HNH domain, cleaves a single strand of DNA.^16^ A double-stranded DNA donor template was constructed consisting of homology arms of ~1 kb flanking a floxed puromycin drug resistance gene in such a way that it would be inserted into an adjacent intron for selection and subsequent cre-recombinase-mediated removal (Figure 1c and Supplementary Figure S2a). To allow for unambiguous determination of donor-derived HDR, we included silent polymorphisms within the right donor arm (Supplementary Figure S2a). The donor fragment and the nuclease or nickase version of Cas9 and gRNA 2 were electroporated into fibroblasts that were then puromycin-selected in bulk. The puromycin-resistant cells were screened for HDR using an ‘inside-out’ PCR strategy, with a primer inside the donor and outside of the right homology arm.

Bulk population cells were plated at low density to allow for clonal selection, expansion and screening, and 17 total clones were obtained. Four clones that received the Cas9 nickase, and eight treated with Cas9 nuclease, showed HDR at the genomic level (Figure 1d and Supplementary Figure S2b). The puromycin cassette was subsequently removed using cre-mRNA, which resulted in a small *loxp* footprint in the intron (Supplementary Figure S2c). Screening at the cDNA level showed gene correction and donor polymorphism presence in *COL7A1* gene transcripts (Supplementary Figure S2d). These data show the ability of the CRISPR/Cas9 to mediate gene repair in primary fibroblasts obtained from an RDEB patient that, importantly, retained a normal morphology and karyotype (Supplementary Figures S3a and b).

An important consideration for the employment of programmable gene-editing reagents is their specificity for the intended gene target. Because of the presence of sequences of partial homology to the *bona fide* target, we utilised a predictive *in silico* modelling algorithm to identify potential off-target sites (Table 1). To determine whether promiscuous CRISPR/Cas9 cutting occurred, we screened the putative off-target sites using the Surveyor assay. We observed one off-target site in the *ACAP3* gene (Supplementary Figure S4) that functions as a GTPase activating protein.^17^ Importantly, the nickase version of Cas9 that preferentially promotes HDR^15^ did not show any mutagenic non-homologous end joining at this locus (Supplementary Figure S4) and we therefore employed nickase-corrected clones for reprogramming to pluripotency.

### RDEB gene-corrected iPSCs

Utilising Sendai virus-based reprogramming, we obtained transgene-free, gene-corrected, karytotypically normal iPSCs (Supplementary Figures S3c and d). Resultant iPSC clones were positive for pluripotent markers at the protein (Supplementary Figure S5) and gene expression levels (Supplementary Figure S6a). As further verification of successful reprogramming, the *OCT4* and *NANOG* gene promoters were observed to be hypomethylated, and *in vivo* teratomas derived from gene-corrected iPSCs contained representative tissues from all three germ layers. (Supplementary Figures S6b and c).

These results demonstrate that CRISPR/Cas9 genome modification and Sendai virus reprogramming allow for precision repair and iPSC generation. We subsequently employed this population of cells to derive therapeutic cell types suitable for cellular therapies for RDEB.

### Production of keratinocytes from gene-corrected iPSCs

**Figure 2:**
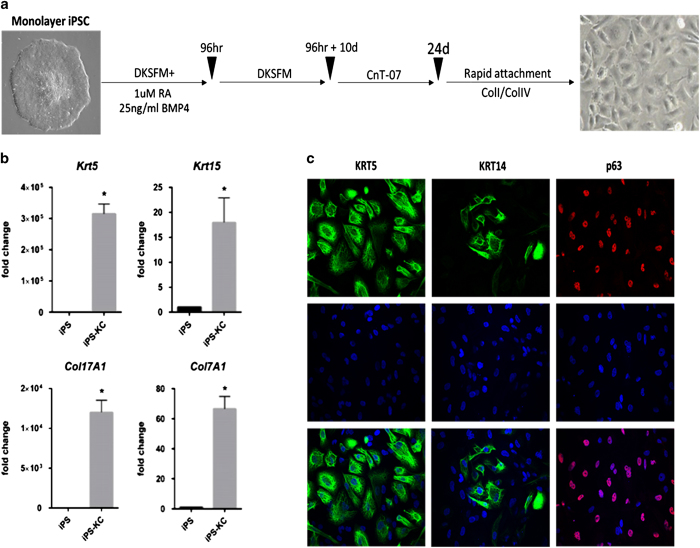
Keratinocyte generation. (**a**) Derivation schema. iPSC colonies in a six-well plate were exposed to RA and BMP-4 in defined keratinocyte serum-free media with a transition to the CnT-07-defined keratinocyte media. Keratinocytes were enriched by exploiting their ability to rapidly attach to collagens I- and IV-coated plates. (**b**) Keratinocyte gene expression analysis. TaqMan RT-qPCR was performed for keratin 5 and 15, *COL17A1* and *COL7A1* (three different experiments) and compared with parental iPSCs. (**c**) Keratinocyte immunofluorescence. Gene-corrected, iPSC-derived cells were stained with anti-KRT5, KRT15 or p63 antibodies (top). Middle panel is DAPI nuclear staining and bottom is merged images that are representative of 3–4 independent experimental replicates. BMP, bone morphogenic protein; RA, retinoic acid; RT-qPCR, quantitative PCR with reverse transcription.

Treatment of the chronic wounds experienced by RDEB patients could be bolstered by the localised delivery of gene-corrected keratinocytes. To demonstrate that iPSCs derived from CRISPR/Cas9 gene-corrected RDEB fibroblasts are capable of differentiating into therapeutically relevant cell populations *in vitro*, we utilised protocols to produce keratinocytes under fully defined, feeder-free conditions.^18–20^ Two-dimensional culture of iPSCs in the presence of retinoic acid (RA) and bone morphogenic protein-4 (BMP-4) resulted in the formation of cells with characteristic epidermal morphology (Figure 2). Compared with undifferentiated iPSCs, differentiation cultures enriched for keratin-expressing epidermal cells by rapid attachment to type IV collagen-coated tissue culture plates exhibited significantly elevated expression of mRNAs associated with commitment to the keratinocyte lineage as well as elevated expression of *Col7A1* mRNA (Figure 2b). Concordant with keratinocyte morphology and mRNA expression profile, iPSC-derived keratinocyte cultures contained a high frequency of cells positive for keratin-5 (Krt5), keratin-14 (Krt14) and transcription factor p63 (Figure 2c). These data show the ability of CRISPR/Cas9 gene-corrected iPSCs to successfully differentiate into keratinocytes *in vitro* under fully defined, xeno-free culture conditions amenable for clinical translation.

### Production of MSCs from gene-corrected iPSCs

**Figure 3:**
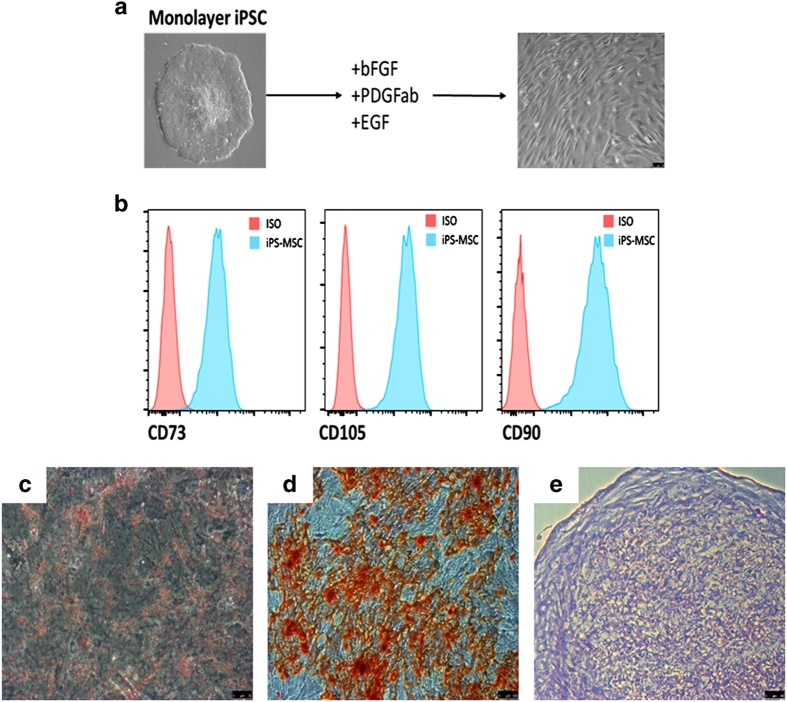
Mesenchymal stem cell derivation. (**a**) MSC differentiation. Mono-layer iPSCs were subjected to bFGF, PDGFab and EGF resulting in differentiation to a cell population with spindle-shaped morphology (right). (**b**) FACS analysis. Passage 3 MSCs were analysed for cell surface expression of CD73, CD105, and CD90 (*n*=3 experiments), and histogram analysis is shown in blue. Isotype antibody control FACS histograms are shown in pink. (**c**,**d**) Tri-lineage differentiation. (**c**) Oil red-O staining demonstrating the ability of iPSC-derived MSCs to form adipose cells. (**d**) Alizarin red staining of osteogenic progeny. (**e**) Toluidine blue staining of chondrogenic cells from MSCs. (**c**–**e**) Representative images of at least two different MSC pools and *n*=3–4 replicates. FACS, fluorescence-activated cell sorting.

MSCs are mesoderm-derived, fibroblastic cells present within many tissues, including bone marrow and adipose. There is substantial evidence that MSCs or MSC-derived cells can enhance wound healing via modulation of wound microenvironment, immunomodulation, or by direct integration into cutaneous tissues after transplant.^21–26^ At present, targeted gene editing in primary human MSCs has not been demonstrated, leading us to pursue directed differentiation of *COL7A1* gene-corrected iPSCs to MSCs *in vitro*.^27^ *COL7A1* gene-corrected iPSCs were exposed to MSC media supplemented with platelet-derived growth factor (PDGF)-AB, basic fibroblast growth factor (bFGF), and epidermal growth factor (EGF) until iPSC colony morphology was lost and outgrowth of cells with fibroblastic morphology was observed (Figure 3a). At this stage the cells were passaged from the matrigel substrate to gelatin-coated plastic tissue culture vessels. We observed that cell survival and re-plating during passages 1–3 is substantially enhanced in the presence of Rho-associated protein kinase (ROCK) inhibitor, allowing for more consistent establishment of robust iPSC-MSC cultures capable of expansion with minimal senescence (Supplementary Figure S7a). Between passages 3–5, uniform iPSC-MSC cultures were obtained and flow cytometric analysis demonstrated the expression of MSC markers CD73, CD90 and CD105 (Figure 3b). Furthermore, we show that *COL7A1*-corrected iPSC-derived MSCs were able to undergo robust tri-lineage differentiation to adipocytes, chondrocytes and osteoblasts *in vitro* (Figures 3c–e); a defining characteristic of primary MSCs.^28^ Collectively, these data show that *COL7A1* gene-corrected iPSCs are capable of producing MSCs *in vitro*, and that these iPSC-MSCs are indistinguishable from primary human MSCs in their morphology, cell surface antigen profile, and their capacity for tri-lineage differentiation and retain the ability to self-renew in culture (Figure 3 and Supplementary Figure S7). Moreover, the gene-corrected iPSC-derived MSCs but not parental uncorrected fibroblasts showed collagen type VII protein expression (Supplementary Figure S7c).

### Production of definitive haematopoietic progenitors from gene-corrected iPSCs

**Figure 4:**
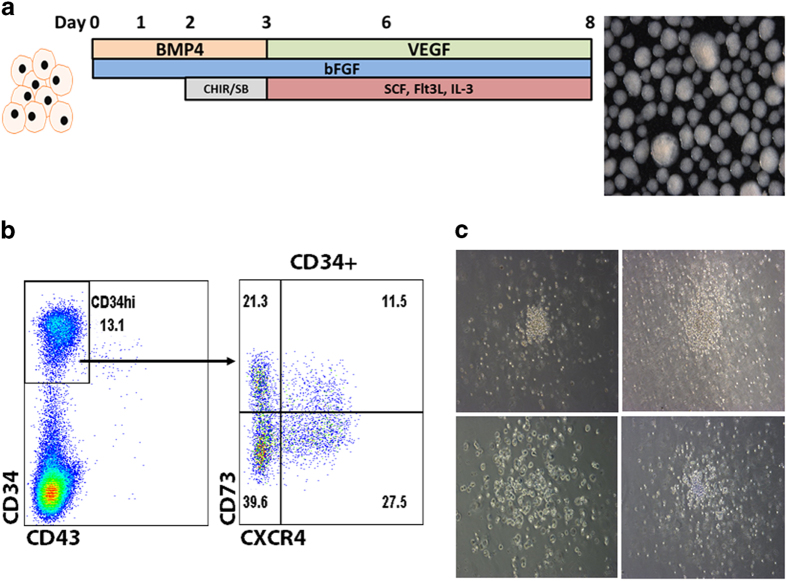
Haematopoietic differentiation. (**a**) Culture condition schema. Embryoid bodies were formed from iPSCs in the presence BMP4, VEGF, bFGF, CHIR99021 and SB431542, and haematopoietic cytokines (Flt3-ligand, SCF and IL-3) over the course of 8 days. A representative image of EBs are shown at right. (**b**) FACS phenotype of EB-derived CD34. Dissociated EBs were analysed by FACS for CD73 and CXCR4 in the CD34 high-expressing population. (**c**) Methylcellulose CFU assay. CD34 cells were embedded in methylcellulose, and an image of the dominating CFU-G/M colony types are shown. The analyses/experiments are from at least four different differentiation procedures. FACS, fluorescence-activated cell sorting.

**Figure 5:**
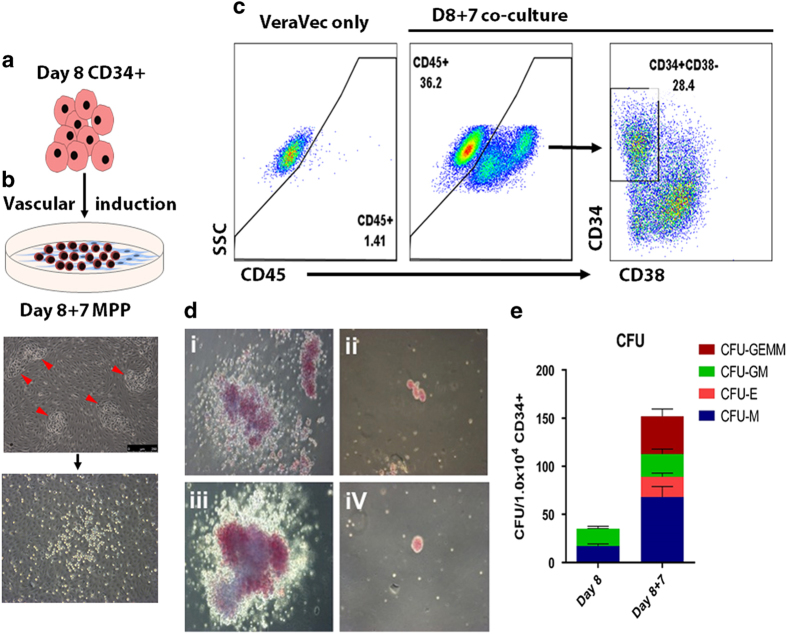
Haematopoietic differentiation and vascular induction. (**a**) Experimental design. EBs formed over 8 days (as shown in this figure), were dissociated, sorted for CD34 cells, and then incubated on the VeraVec endothelial cell line expressing the *E4ORF1* gene. (**b**) Multipotent haematopoietic progenitors. Upon vascular induction, small clusters of budding hemogenic cells (red triangles) were evident by light microscopy and gave rise to non-adherent haematopoietic progenitors. (**c**) FACS phenotype of iPSC CD34s that underwent vascular induction. Day 8 CD34 from EBs cultured on VeraVec cells for 1 week was analysed by flow cytometry for CD34, 48 and 45. (**d**) Vascular induction CFU. CD34 CD45+ cells isolated from the VeraVec feeder cells were cultured in methylcellulose, and representative colonies for CFU-GEMM (i and iii) and CFU-E (ii and iv) are shown. (**e**) CFU quantification. Three independent experiments were analysed and quantified for the number of lineage-specific colonies formed for CD34 cells derived +/− vascular induction. CFU-GEMM, colony-forming unit-granulocyte, erythrocyte, monocyte/macrophage, megakaryocyte; CFU-GM, colony-forming unit granulocyte- monocyte/macrophage; CFU-E, colony-forming unit erythroid; CFU-M, colony-forming unit monocyte/macrophage; FACS, fluorescence-activated cell sorting.

Although cutaneous blistering is the most apparent pathology observed in patients with RDEB, it is the systemic manifestations—including mucosal blistering—that are often the most destructive and life-threatening and which cannot be resolved by localised grafting of epidermal cell types such as keratinocytes or fibroblasts. Our group and others have previously reported the amelioration of systemic RDEB pathology by HCT that is associated with a substantial risk of morbidity and mortality during the preparative and post-transplant period.^29,30^ Therefore, the generation of gene-corrected HSPCs would be a highly desirable approach for autologous therapy. Although targeted gene-editing in severe combined immune deficiency-repopulating human HSPCs has been demonstrated, the efficiency is not yet robust enough for models such as RDEB that do not confer a selective or proliferative advantage to the modified cell(s).^31–33^ Therefore, we undertook efforts to advance the understanding of *in vitro* HSPC generation using a translational workflow under serum and feeder-free conditions. Protocols for serum-free haematopoietic differentiation are well described, yet in each of these reports the starting iPSCs are cultured on xenogeneic murine feeder cells, which is suboptimal for clinical translation.^34–36^ Our efforts to implement these protocols to feeder-free iPSC cultures were met with limited success, and we observed that embryoid body formation, growth and production of CD34+ hemogenic progenitors was poor (Supplementary Figure S8). In light of these observations we sought to optimise the differentiation protocol to allow robust generation of haematopoietic progenitors under fully defined, serum-free, xeno-free conditions. We employed the nutrient-rich, human serum albumin-based media TeSR1 for maintenance of feeder-free cells, and generated embryoid bodies (EB) in the presence of the haematopoietic progenitor medium StemPro34. Haematopoietic induction frequency is highest when cells transit through an EB stage, and we observed that the abrupt transition from TeSR1 to StemPro34 resulted in poor haematopoietic cell derivation (Supplementary Figure S8). Therefore, we pursued EB formation in StemPro34 supplemented with dilutions of TeSR1 and found that EB formation was enhanced substantially (Supplementary Figure S8). In support of our ultimate goal of feeder-free iPSCs with differentiation carried out in fully defined media, we sought to identify the conditions for elimination of TeSR1 media that contains undefined human serum albumin. We therefore employed animal-free polyvinyl alcohol essential lipid (APEL) as a base media that allowed for sustained growth of EB after TeSR1 removal and robust production of CD34+ haematopoietic progenitors at day 8 (Figure 4a).^37^ As part of this procedure we modulated the commitment phase of blood development by combining Activin/Nodal inhibition and GS3Kβ inhibition at the mesoderm phase of differentiation in order to bias the system towards definitive haematopoiesis.^38^ A substantial portion of the CD34+ fraction at day 8 was negative for the mesenchymal marker CD73 and the vascular marker CXCR4, representing a robust population of hemogenic precursors (Figure 4b).^34^ We further confirmed the definitive potential of EB-derived CD34+ cells via demonstration of T-lineage specification upon co-culture with OP9-DLL4 (Supplementary Figure S9).^34–36,39^ However, when assessed directly *in vitro* by methylcellulose-based haematopoietic colony-forming unit (CFU) assay, the day 8 EB CD34+ fraction exhibited a markedly limited myeloid potential, indicating that further maturation was required to complete ultimate specification to committed HSPCs (Figure 4c). Given the complex niche in which definitive HSPCs arise during development, we next sought to recapitulate that environment *in vitro*. To accomplish this we employed a supportive human vascular stromal endothelial cell population that expresses the adenovirus *E4ORF1* gene and has been shown to support endothelial to HSPC derivation *in vitro*.^40,41^ We generated EB-derived mature CD34+ cells as before (Figure 5a), and at day 8 purified CD34+ hemogenic precursors were co-cultured with the vascular stroma and subsequently underwent endothelial to haematopoietic transition to form rounded haematopoietic cells that contained a subpopulation of CD45+CD34+CD38- haematopoietic progenitors (Figures 5b and c). In CFU assays, day 8 EB-derived CD34+ cells that underwent vascular induction showed a broader colony differentiation potential (Figure 5d). In aggregate, day 8 EBs with 7-day co-culture on a supportive vascular endothelial matrix resulted in both a significant increase in colony frequency and an expansion in multilineage potential compared with day 8 EBs alone (Figure 5e). These data demonstrate that *COL7A1*-corrected iPSCs represent a platform for the *in vitro* production of definitive haematopoietic progenitors capable of producing multiple blood lineage cell types in CFUs that represent an advance towards true HSPC *in vitro* generation.

## Discussion

RDEB is associated with a loss of the functional integrity of the dermal-epidermal junction, which results in painful erosions and blistering. The disease process is not limited to the cutaneous manifestations; therefore, a platform for combinatorial regenerative medicine must possess attributes of tissue plasticity, migratory properties and an effector lineage capable of C7 deposition. To date, infusions of allogeneic HSCs and progenitor cells, fibroblasts, keratinocytes and MSCs have all been pursued clinically. Although HCT confers a lifelong supply of donor-derived cells, the mechanism for wound repair remains poorly understood. Further, areas of the skin are often refractory to benefit from HCT, and supportive localised therapy is required for more penetrant effects. Fibroblasts and keratinocytes are the primary C7-producing cells in the skin and have been utilised for localised, supportive therapy as have MSCs, which possess the capacity to home to wounded tissue and deposit C7.^42^ No one cell type is endowed with each of these beneficial properties, and post-injection cellular persistence is transient in nature requiring repetitive delivery necessitating a manufacturing process reliant on long-term culture and expansion that may drive the cells towards senescence. Autologous RDEB iPSCs that are derived from patient cells represent a potential solution to this shortcoming provided that a corrected *COL7A1* gene sequence is present.^18,43^ To maximise the potential of this approach, we coupled cellular reprogramming with precision gene correction, representing a platform for production of autologous cell types for regenerative medicine.^44–47^

Members of our group,^44^ and Chamorro *et al.*^47^ have utilised transcription activator-like effector nucleases for *COL7A1* gene correction in fibroblasts and keratinocytes, respectively. In a study by Izmiryan *et al.* meganucleases with an integrase-deficient lentiviral vector were used to mediate gene repair in keratinocytes and fibroblasts.^45^ Sebastiano *et al.*^46^ and Wenzel *et al.*^48^ used iPSCs as a template for gene correction without classically engineered nucleases. They used a flip recombinase in murine cells or a pro-recombinogenic corrective donor construct alone delivered on an adeno-associated viral vector in human cells, respectively.^46^ These studies show that the RDEB genotype and phenotype are amenable to correction by genome engineering. Building off these studies we set out to resolve two gaps in the current RDEB cellular and genome-engineering procedures: (i) integration-free derivation and subsequent feeder-free maintenance of iPSCs; and (ii) the utilisation of genetically corrected iPSCs for generation of multiple therapeutic cell types under defined conditions as proof of concept for multilineage cellular therapy (Figure 1). Towards the former, we and others have used integrating vector delivery of the iPSC reprogramming factors that either remain in the genome or require subsequent removal.^43,44^ In the latter, our previous work relied on *in vivo* teratoma formation that occurs in poorly defined conditions that are not able to be controlled *ex vivo*.^44^ Sebastiano *et al.* report efficient generation of minimally heterogeneic keratinocytes; however, this cell population does not represent a durable modality for broad use. As such, in the current work we describe a gene correction strategy employing the CRISPR/Cas9 system that resulted in gene correction in fibroblasts that are of immediate value for localised C7 production. We then employed the Sendai reprogramming methodology to generate iPSCs that were used for subsequent derivation of multilineage cell types with therapeutic value.

Three CRISPR/Cas9 targeting candidates were tested, with one employed for gene correction in comparative studies with the nuclease or nickase version of the *S. pyogenes* Cas9 (Supplementary Figure S1). Similar to previous observations, we observed a lower overall rate of HR using the nickase (4/12 clones showing HDR versus 8/12 for the nuclease); however, the preferential repair of DNA nicks by the HR pathway adds a further layer of specificity to the engineering process. This is highlighted by the observation of an off-target event at the *ACAP3* gene with the nuclease, but not nickase, version of Cas9 (Table 1 and Supplementary Figure S4). The derivation of clones was facilitated by our donor design strategy that included a puromycin cassette that was knocked into an adjacent intron for subsequent removal by cre-recombinase (Figure 1 and Supplementary Figure S2). This approach allowed us to obtain a homogenous population of fibroblasts by selection, as opposed to non-selection-based strategies that have observed gene correction rates of <10%.^45^ This consideration is crucial given that the subsequent reprogramming process results in a sub-fraction of cells reprogrammed to pluripotency and, if the input is not uniform, extensive screening is mandated. Our target cell population was carefully considered due to the fact that fibroblasts competent for C7 protein expression have utility for localised therapy in RDEB.^49^ Given the comparatively diminished proliferation capacity and rate of fibroblasts compared with iPSCs, we also attempted gene correction in iPSCs due to the fact that HDR occurs in late S and G2.^50,51^ Surprisingly, we were unable to achieve gene correction in this patient’s iPSCs, while we obtained 12 corrected fibroblast clones. One possible explanation for this is poor accessibility due to the chromatin status of iPSCs versus fibroblasts. Given that at the resolution of western blotting we were unable to show C7 protein expression in iPSCs (Supplementary Figure S7c), and the poor ability of nucleases to modify silent or repressed genes,^52^ we postulate that the 4317 position of the *COL7A1* locus may be refractory to gene modification as a result of chromatin state.

The Sendai virus reprogramming method is a non-integrating RNA virus platform that mediates robust reprogramming frequencies without retaining the reprogramming factors by virtue of natural loss by dilution as the cells divide.^53^ As such, the need for secondary factors (e.g., cre-recombinase) to remove the viral footprint is eliminated, as is the risk for adverse events associated with the random integration of viral vectors. Using this methodology, we obtained numerous clones and chose two (11-2 and 11-5) for iPSC quality assurance and control assessment. Each clone exhibited morphology consistent with pluripotency (i.e., discrete colonies with rounded edges), were karyotypically normal (Supplementary Figure S3), and expressed pluripotency-associated markers NANOG, TRA-60, −81, SSEA-3, −4 and OCT-3/4 (Supplementary Figure S5). Transcriptional profiling and promoter methylation analysis further confirmed successful reprogramming, and iPSC clones implanted into immune-deficient animals gave rise to teratomas consisting of tissues representative of all three germ layers (Supplementary Figure S6). These data demonstrate the successful reprogramming of CRISPR/Cas9 gene-corrected fibroblasts to iPSCs, which were subsequently maintained and propagated in feeder-free conditions and served as a renewable template for the generation of keratinocytes, MSCs and HPSCs.

Using defined conditions, we were able to derive epidermal cells with a morphology consistent with that of keratinocytes, and these cells expressed the keratinocyte markers KRT5, KRT14, p63, COL17A1 and COL7A1 (Figure 2). These iPSC-derived keratinocytes hold promise for localised application to chronic and/or severe wounds—either alone or in support of systemic therapy.

MSCs have shown promise as both a localised and systemic therapeutic intervention in the setting of RDEB. However, genetic correction of MSCs has not been demonstrated, leading us to pursue *in vitro* production of MSCs from our gene-corrected iPSCs. By exposing iPSCs to bFGF, PDGF and EGF, and utilising ROCK inhibition during the early passages, we were able consistently produce robust MSC cultures (Supplementary Figure S7). These cells were morphologically identical to bone marrow-derived MSCs (Figure 3a and Supplementary Figure S7), and were able to be expanded and propagated over multiple passages while retaining surface expression of CD73, CD105 and CD90, and the ability to undergo chondro-, osteo- and adipo-genic differentiation in a manner identical to bone marrow-derived MSCs (Figures 3b–e and Supplementary Figure S7). Importantly, from ~5×10^6^ starting iPSCs, we were routinely able to generate >1×10^8^ iPSC-MSCs (data not shown). Considering that a recent clinical trial using systemic delivery of MSCs for treatment of RDEB reported a dose of 1–3×10^6^/kg^3^, our protocol is capable of generating clinically relevant yields of iPSC-MSCs.

A highly challenging hurdle in stem cell biology remains the *in vitro* conversion of iPSCs to definitive HPSCs. Despite substantial effort, there remains no robust, efficient, or clinically translatable method for converting iPSCs into HPSCs capable of long-term engraftment. Towards this goal, we sought to synergise several of the most promising advancements in this area to achieve a more robust platform for producing definitive-type HPSCs. The first improvement involved optimising the basal media composition for robust production and growth of EBs from feeder-free iPSCs under fully defined conditions. This centred on the stepwise induction of mesoderm with BMP-4 and bFGF followed by specification to haematopoiesis with vascular endothelial growth factor, stem cell factor, IL-3 and Flt-3 ligand. Further steps were small molecule inhibition of the Activin/Nodal pathway with SB431542 with augmentation of the Wnt pathway^36^ via inhibition of GS3Kβ with CHIR99021 to drive definitive haematopoiesis (Figures 4 and 5 and Supplementary Figure S9). Our hypothesis was that the culture conditions and modulation of the primitive and definitive fate determinant modulators with CHIR99021 and SB431542 would bias the cells away from primitive haematopoiesis and promote definitive haematopoietic commitment. However, even though EB-derived CD34+ hemogenic precursors were definitive in nature as evidenced by their capacity for T-lineage differentiation given the appropriate micro environmental cues, their intrinsic capacity for multilineage haematopoietic differentiation was limited in CFU assays. (Figure 4 and Supplementary Figure S9c). This prompted us to employ a vascular induction technique aimed at recapitulating the complicated embryonic haematopoietic niche environment *ex vivo,* to determine whether the instructive cues provided by the *E4ORF1* transformed endothelial cells would promote maturation and expansion of EB-derived CD34+ cells to multilineage haematopoietic progenitors. In contrast to the data observed when EBs were utilised without vascular induction, the inclusion of the co-culture system resulted in the substantial increase of both the progenitor frequency and multilineage capacity (Figures 4 and 5). These data outline a robust, reproducible strategy for the *in vitro* production of multilineage definitive HPSCs from iPSCs.

In conjunction with our corresponding data showing the ability to correct RDEB-causing mutations and employ an engineering strategy that maximises the potential of iPSCs for therapeutic cell generation these results hold great promise for RDEB and other maladies that may benefit from regenerative medicine.

## Materials and methods

### Research subject and cell line derivation

Patient-derived samples were obtained following parental consent and approval from the University of Minnesota Institutional Review Board. A 4 mm skin punch biopsy was collected followed by mincing the skin tissue and submerging in complete DMEM media with 20% FBS, non-essential amino acids, antibiotics and glutamax all from Invitrogen, (Carlsbad, CA, USA). Primary fibroblasts were maintained in complete DMEM media under hypoxic (<2% O_2_) conditions.

### Mice

NOD/SCID IL2rγcnull (NSG) mice were ordered from Jackson Laboratories (Bar Harbor, ME, USA). All animal studies were approved by the University of Minnesota Institutional Animal Care and Use Committee.

### CRISPR/Cas9 reagent and donor construction

The *S. pyogenes* hCas9 plasmid was a gift from Dr George Church (Addgene plasmid #41815),^54^ and the gRNAs were assembled into a plasmid with a U6 promoter and polIII termination signal.^15^ The donor sequence was comprised of left and right arms of homology that were assembled by amplification from the human genome with: Left arm F: 5′-CCTGACCTCTTCACCTCCTCAGGGCTTCC-3′, Left Arm R: 5′-GGGCCACACCTCACTCCCAAAGATACCAGG-3′. The Right arm was amplified with RT Arm1 F: 5′-AGGGTCATGGGGTCGTCATCTGTTTTCTAGGG-3′ and Reverse: 5′-AACTATGAAGCCCAGCACCCAACCACTGCCCCAGG-3′ that overlapped with a synthesised fragment containing the corrective base and two silent polymorphisms 5′-CTCTCCTGGGGCAGTGGTTGGGTGCTGGGCTTCATAGTTCTTGCTCATATTTTTACTCACTTCTTCCTAGGGTCTTCCTGGCAGCCCTGGACCCCAAGGCCCCGTTGGCCCCCCTGGAAAGAAAGGAGAAAAAGTAGGAAGGCTGACTTGATGATGTCCCAGTTCTGGGGTGGGAGGCTGCGTGCTGGGGGCAGCCTCCCTTCGGTCTTCCCACCCGTGTGTTTCTCCTTCAGGGTGACTC-3′. The remainder of the right donor arm was amplified with: Right Arm2 F: 5′-CACCCGTGTGTTTCTCCTTCAGGGTGACTC-3′ and reverse: 5′-GGGCAAGAAGTCAGAACCAGAAAGGGCACAGC-3′. These fragments were assembled into a plasmid containing the left donor arm followed by a floxed PGK puromycin cassette by Gibson assembly to complete the donor.^55^

### Gene transfer

Primary fibroblasts (200,000) were electroporated with 1 μg each of the Cas9 and gRNA plasmids and 5 μg of the donor using the following settings on the Neon Transfection System (Invitrogen): 1500 V, 20 ms pulse width, and a single pulse.^44^

### Surveyor nuclease

Genomic DNA was isolated 48 h after Cas9/gRNA electroporation and amplified for with Surveyor 13F (5′-CCATGACCCTCATCACTCCT-3′) and Surveyor 708R primers (5′-TTTGGGGGTTCAGAGATTTG-3′) and incubated with the Surveyor nuclease (Integrated DNA Technologies, Coralville, IA, USA)^56^ and resolved on a 10% TBE PAGE gel (Invitrogen).

### Off-target analysis

293T cells were transfected using Lipofectamine 2000 and the Cas9 nuclease or nickase (500 ng) and guide RNA plasmid (500 ng). Genomic DNA was isolated 72 h post gene transfer and PCR amplified with 4317 131F: 5′-TCCCAAAGTCCTTGAAATCC-3′) and 4317 777R: 5′-GCCCACCATATTCAGAATCC-3′) for on-target site amplification. Off-target sites were identified using the MIT CRISPR Design Tool (http://crispr.mit.edu/) and were amplified with following primers: ACAP3F: 5′-ACGGCCTTGTACAGAACTGG-3′, ACAP3R: 5′-GTGCTTTCGCTCCATCTCAC-3′, GRK6F: 5′-CCAGAGGAGCCTTGAGTTTG-3′, GRK6R: 5′-CTACCCAGCCCCCTTACTTC-3′, E2F2F: 5′-TGGTACGTCGAGGGTCCTAA-3′, E2F2R: 5′-CCTTGGAGGCTACTGACAGC-3′, SEC23AF: 5′-GCTACCTCTCCTCCCTCCTC-3′, and SEC23AR: 5′-CCACCGTTTTCCACATCTTT-3′, CARD10F: 5′-GGCTCATCCGTAACCTGCTA-3′, CARD10R: 5′-GGGCAACCTGGAGATACAGA-3′, SYTL1F: 5′-TTTTGTCGAGATGGGGTCTC-3′, SYTL1R: 5′-GGGGACAGTGCATAATCTGG-3′, FADS3F: 5′-AGATGAACCACATCCCCAAG-3′, FADS3R: 5′-TGGACAAGGGTAGGCATAGG-3′, FAM3DF: 5′-AAGAATCAGGAAGCCCAGGT-3′, FAM3DR: 5′-GTCTCAAACAGCCCAGCTTC-3′, MLLT1F: 5′-GAGACCAAGCTGGAAAGCAC-3′, MLLT1R: 5′-AGCTCAGAACCTCAGGACCA-3′, MYO1EF: 5′-CATTCCTCTCTGCCACCTTC-3′, MYO1ER: 5′-TGTTCGCCGATTCCTTTATT-3′, TIE1F: 5′-AGAGGTGACACAGCCCTCAT-3′, TIE1R: 5′-AGGGTCTTCTCCCAGTCAGG-3′, SHANK2F: 5′-CTTTGGGTCCCTGTTGAGAC-3′, SHANK2R: 5′-GAAGACGTGCTCCATCCCTA-3′. PCR conditions were: 94 ° C×2 min followed by 40 cycles of 94 ° C×40 s, 58 ° C×40 s, and 68 ° C×1 min with AccuPrime DNA polymerase (ThermoFisher Scientific, Waltham, MA, USA). PCR products were denatured and renatured and assayed by Surveyor nuclease (Integrated DNA Technologies) and subsequent resolution on a 10% TBE PAGE gel (ThermoFisher Scientific).^15,56^ All gel images used the same exposure times.

### Selection and HDR analysis

80% confluent fibroblasts that had undergone electroporation were exposed to 0.2 μg/ml puromycin and then plated at low density for clonal derivation. Individual cells were segregated in cloning disks and expanded for HDR analysis using an inside-out PCR. A primer inside the donor (5′-GCCACTCCCACTGTCCTTTCCT-3′) and outside the right homology arm (5′-GGGCAAGAAGTCAGAACCAG-3′) were amplified, cloned into the pCR 4 TOPO vector (Invitrogen) and sequenced by the Sanger method to confirm HDR. cDNA correction was demonstrated by amplification of a product with cDNAF: 5′-GTGACAAAGGCGATCGTG-3′ and cDNAR: 5′-GTCCCCGTGGGCCCTGC-3′ followed by sequencing.

### PGK puromycin removal

Cre-recombinase mRNA (TriLink Biotechnologies, San Diego, CA, USA) was electroporated into iPSC clones at a dose of 1 μg using the conditions noted above, and excision was confirmed by PCR and Sanger sequencing.

### iPSC generation and teratoma assay

Gene-corrected fibroblasts (or uncorrected cells as a control) were reprogrammed to iPSCs as described^43,57^ with Sendai virus delivery of the reprogramming factors.^53^ Karyotype, gene expression and immunofluorescence as part of quality assurance and control were also performed as detailed elsewhere.^43,57^ All iPSC lines were regularly tested for mycoplasma and all lines used in this study tested negative. iPSCs contained in matrigel were implanted onto the hind flank of NSG mice (*n*=3–5) until a palpable mass formed. Teratoma tissue was excised for histological examination following embedding and staining by haematoxylin and eosin.

### Differentiation of iPSCs to keratinocytes

iPSCs were maintained feeder-free on Geltrex-coated (ThermoFisher Scientific) tissue culture plastic in TesR1 (STEMCELL Technologies, Vancouver, BC, Canada). For keratinocyte differentiation, mid-passage iPSCs at ~50% confluency in six-well plates had media changed to defined keratinocyte-SFM media supplemented with 25 ng/ml BMP-4 (Bio-Techne, Minneapolis, MN, USA) and 1 μM RA (STEMCELL Technologies) for the first 96 h, at which point the BMP4 and RA were removed, followed by media changes every 72 h thereafter until epithelial cell morphology became apparent (~10 days). At this timepoint, the media was switched to Cnt-07 (CELLnTEC, Bern, Switzerland) and the cells cultured until confluency. At this point they were pre-treated with ROCK inhibitor (Y-27632, VWR) for at least 1 h and passaged using Accutase (STEMCELL Technologies) onto 10 cm^2^ tissue culture plates coated with collagen I/collagen IV, where rapid attachment-mediated enrichment of Krt14+ cells was performed as previously described. Resultant iPSC-keratinocyte cultures were cultured in Cnt-07 media containing 10 μM ROCK inhibitor until first media change after plating (CELLnTEC).

### Differentiation of iPSCs to MSCs

iPSCs were maintained feeder-free on Geltrex-coated tissue culture plastic in TesR1 (STEMCELL Technologies). Differentiation to MSCs was initiated by transferring mid-passage iPSCs at 50% confluency to MSC medium, which consisted of Minimal Essential Medium Alpha supplemented with 5% fetal bovine serum, 5% horse serum and 10 ng/ml each of human PDGF-AB, EGF, and bFGF (all from PeproTech, Rocky Hill, NJ, USA). Media was changed every 48 h until cells with fibroblastic morphology were apparent and cultures neared confluency. At this point, MSC cultures were pre-treated with 10 μM ROCK inhibitor for at least 1 h and dissociated using a 50:50 mixture of Accutase and 0.25% Trypsin-EDTA incubated at 37 ° C. When cells had detached, the Accutase/trypsin mixture was diluted with 2× volume of PBS +1% FBS +3 mM EDTA to prevent clumping. Cells were centrifuged at 400*g* for 5 min and re-plated onto gelatin-coated plates in growth media plus cytokines containing 5 μM ROCK inhibitor to enhance plating efficiency. Between passages 3–5, cultures can be weaned off of ROCK inhibitor during passage. Flow cytometry analysis was performed using the following antibodies, all from eBioscience (San Diego, CA, USA): Anti-Human CD73 eFluor 450, 48-0739; anti-Human CD105 (Endoglin) PE, 12-1057; and anti-Human CD90 (Thy-1) APC, 17-0909.

### Maintenance and differentiation of human iPSCs to haematopoietic progenitors

Human iPSC lines were maintained on Matrigel- or Geltrex-coated plastic ware in TesR1 (STEMCELL Technologies). For differentiation, hiPSCs were cultured at ~80–90% confluency, followed by EB generation, as described previously.^37,38^ Briefly, the undifferentiated hiPSCs were dissociated with Accutase (STEMCELL Technologies) treatment. Aggregates were resuspended in APEL-differentiation medium (STEMCELL Technologies), supplemented with BMP-4 (20 ng/ml) and bFGF (5 ng/ml) and cultured in non-tissue culture treated plates. After 42 h, developing EBs were collected and resuspended in APEL-differentiation media supplemented with BMP-4, bFGF, CHIR99021 (3 μM, Stemgent, Lexington, MA, USA), and SB431542 (6 μM, Selleck Chemicals, Houston, TX, USA).

After 24 h, EBs were collected and resuspended in either 60% APEL-differentiation medium +40% STEMSPAN II medium (STEMCELL Technologies) for vascular induction, or 100% APEL-differentiation medium for T-lineage differentiation; both supplemented with VEGF (20 ng/ml), bFGF (5 ng/ml), IL-3 (20 ng/ml), Flt3L (20 ng/ml) and SCF (100 ng/ml) and cultured for another 5–6 days. Cultures were maintained in a 5% CO_2_/5% O_2_/90% N_2_ environment. At day 8 or 9, EBs were harvested, washed once with PBS, and dissociated using Accutase and 0.25% trypsin EDTA mixture until no visible clumps observed. CD34+ cells were enriched using Easy-Sep CD34+ isolation kit (STEMCELL Technologies). Flow cytometry analysis was performed using the following antibodies, all from eBioscience: Anti-Human CD45 APC-eFluor 780, 47-0459; anti-Human CD34 APC, 17-0349-42; anti-Human CD43 PE, 12-0439; anti-Human CD73 eFluor 450, 48-0739; anti-Human CD184 (CXCR4) PE-Cy7, 25-9999-42.

### Vascular induction

For vascular induction, 1×10^5^ purified EB-derived CD34+ cells were plated onto 85% confluent VeraVec HUVEC (Angiocrine Bioscience, San Diego, CA, USA) in StemSpan II supplemented with the following: SCF (200 ng/ml), Flt3L (10 ng/ml), TPO (30 ng/ml), IL-11 (5 ng/ml), IGF-1 (25 ng/ml), bFGF (5 ng/ml), VEGF (5 ng/ml), EPO (2 IU/ml), IL-6 (10 ng/ml), IL-3 (30 ng/ml), BMP-4 (10 ng/ml) and losartan (100 μM). Co-cultures were maintained for 7–9 days.

### T-lineage differentiation

For T lineage differentiation, 1×10^5^ purified CD34+ cells were plated onto confluent OP9-DLL4 cells for about 3–4 weeks and passaged every 4–5 days as described previously.^35^ All recombinant factors are human and were purchased from R&D Systems (Minneapolis, MN, USA).

### Colony-forming unit assay

Cells were placed in MethoCult according to the manufacturer’s instructions (STEMCELL Technologies). Colonies were enumerated by an experienced analyst using light microscopy at low magnification (×4 objective).

### Western blotting

Cellular lysates in RIPA lysis buffer (ThermoFisher Scientific) were electrophoresed using the XCell SureLock Mini-Cell Electrophoresis System with a NuPAGE Novex 3–8% acetate gel (ThermoFisher Scientific). Proteins were transferred to a nitrocellulose membrane and stained with the 4D2 mouse monoclonal anti-human collagen 7 antibody (Santa Cruz Biotechnology, Dallas, TX, USA). Secondary staining was performed with a goat anti-mouse horseradish peroxidase conjugated antibody (Santa Cruz Biotechnology) and detection with the SuperSignal West Pico Chemiluminescent Substrate (ThermoFisher Scientific).

### Statistics

Statistical differences in survival were determined by Log-rank test using Prism software (GraphPad Software, La Jolla, CA, USA). All other statistical analyses were performed using the Student's t-test with *P* values less than 0.5 being considered significant. No statistical methods were used to predetermine the sample sizes. The experiments were not randomized and the investigators were not blinded.

## Figures and Tables

**Table 1:** Off-target activity analysis

*Target*	*Gene*	*Function*	*Surveyor (nuclease/nickase)*
GGAGGCTGCGTGCTGGGGGC AGG	*COL7A1*	Anchoring fibril	+/−
**AC**AGGCTGC**A**TG**T**TGGGGGC **T**GG	*ACAP3*	GTPase activation	+/−
GG**G**GGC**CT**CG**G**GCTGGGGGC **TA**G	*GRK6*	G protein kinase	−/−
GG**G**GGC**A**G**GC**TGCTGGGGGC AGG	*E2F2*	Transcription factor	−/−
GG**C**GGC**G**GCG**G**GCTGGGGGC **T**GG	*SEC23A*	Zinc ion binding	−/−
GGA**A**G**G**TG**G**GTGCTGGGGGC **T**GG	*CARD10*	Apoptosis signalling	−/−
**T**G**G**GGCT**A**CGT**C**CTGGGGGC **CA**G	*SYT11*	Exocytosis/transport	−/−
**T**GAG**TG**TG**G**GTGCTGGGGGC **CA**G	*FADS3*	Lipid metabolism	−/−
GGAGG**T**TG**G**G**G**GCTGGGGGC **T**GG	*FAM3D*	Insulin secretion	−/−
GG**T**G**AG**TG**A**GTGCTGGGGGC AGG	*MLLT1*	DNA binding	−/−
**A**G**G**GGCTG**G**G**G**GCTGGGGGC **T**GG	*MYO1E*	ATP binding	−/−
**T**G**G**GGCTG**G**G**C**GCTGGGGGC **CA**G	*TIE1*	ATP binding	−/−
G**AG**GGC**C**GCGT**C**CTGGGGGC **G**GG	*SHANK2*	Synaptic protein	−/−

CRISPR Design Tool-identified intragenic off-target sites were assessed. The mismatches between the *COL7A1* target site and the putative off-target sites are in bold. Target genes and their functions are shown. At right are the results of the Surveyor analysis in Cas9 nuclease- or nickase-treated cells. The +/− sign indicates whether locus modification as assessed by the Surveyor assay was observed or not.

## References

[bib1] van den Akker, P. C. et al. The International Dystrophic Epidermolysis Bullosa Patient Registry: an online database of dystrophic epidermolysis bullosa patients and their COL7A1 mutations. Hum. Mutat. 32, 1100–1107 (2011). 21681854 10.1002/humu.21551

[bib2] Wagner, J. E. et al. Bone marrow transplantation for recessive dystrophic epidermolysis bullosa. N. Engl. J. Med. 363, 629–639. 20818854 10.1056/NEJMoa0910501 PMC2967187

[bib3] Petrof, G. et al. Potential of systemic allogeneic mesenchymal stromal cell therapy for children with recessive dystrophic epidermolysis bullosa. J Invest Dermatol 135, 2319–2321 (2015). 25905587 10.1038/jid.2015.158 PMC5696540

[bib4] Latifi-Pupovci, H. et al. *In vitro* migration and proliferation ('wound healing') potential of mesenchymal stromal cells generated from human CD271(+) bone marrow mononuclear cells. J. Transl. Med. 13, 315 (2015). 26407865 10.1186/s12967-015-0676-9 PMC4582892

[bib5] Wagner, J. E. et al. Bone marrow transplantation for recessive dystrophic epidermolysis bullosa. N. Engl. J. Med. 363, 629–639 (2010). 20818854 10.1056/NEJMoa0910501 PMC2967187

[bib6] Petrof, G. , Martinez-Queipo, M. , Mellerio, J. E. , Kemp, P. & McGrath, J. A. Fibroblast cell therapy enhances initial healing in recessive dystrophic epidermolysis bullosa wounds: results of a randomized, vehicle-controlled trial. Br. J. Dermatol. 169, 1025–1033 (2013). 24032424 10.1111/bjd.12599

[bib7] Woodley, D. T. et al. Intradermal injection of lentiviral vectors corrects regenerated human dystrophic epidermolysis bullosa skin tissue *in vivo*. Mol. Ther. 10, 318–326 (2004). 15294178 10.1016/j.ymthe.2004.05.016

[bib8] Georgiadis, C. et al. Lentiviral engineered fibroblasts expressing codon-optimized *COL7A1* restore anchoring fibrils in RDEB. J. Invest. Dermatol. 136, 284–292 (2016). 26763448 10.1038/JID.2015.364 PMC4759620

[bib9] Hacein-Bey-Abina, S. et al. Insertional oncogenesis in 4 patients after retrovirus-mediated gene therapy of SCID-X1. J. Clin. Invest. 118, 3132–3142 (2008). 18688285 10.1172/JCI35700 PMC2496963

[bib10] Christian, M. et al. Targeting DNA double-strand breaks with TAL effector nucleases. Genetics 186, 757–761 (2010). 20660643 10.1534/genetics.110.120717 PMC2942870

[bib11] Porteus, M. H. & Carroll, D. Gene targeting using zinc finger nucleases. Nat. Biotechnol. 23, 967–973 (2005). 16082368 10.1038/nbt1125

[bib12] Paques, F. & Duchateau, P. Meganucleases and DNA double-strand break-induced recombination: perspectives for gene therapy. Curr. Gene Ther. 7, 49–66 (2007). 17305528 10.2174/156652307779940216

[bib13] Cong, L. et al. Multiplex genome engineering using CRISPR/Cas systems. Science 339, 819–823 (2013). 23287718 10.1126/science.1231143 PMC3795411

[bib14] Shen, B. et al. Efficient genome modification by CRISPR-Cas9 nickase with minimal off-target effects. Nat. Methods 11, 399–402 (2014). 24584192 10.1038/nmeth.2857

[bib15] Osborn, M. J. et al. Fanconi anemia gene editing by the CRISPR/Cas9 system. Hum. Gene Ther. 26, 114–126 (2015). 25545896 10.1089/hum.2014.111 PMC4326027

[bib16] Bannwarth, S. , Procaccio, V. & Paquis-Flucklinger, V. Surveyor nuclease: a new strategy for a rapid identification of heteroplasmic mitochondrial DNA mutations in patients with respiratory chain defects. Hum. Mutat. 25, 575–582 (2005). 15880407 10.1002/humu.20177

[bib17] Stelzl, U. et al. A human protein-protein interaction network: a resource for annotating the proteome. Cell 122, 957–968 (2005). 16169070 10.1016/j.cell.2005.08.029

[bib18] Itoh, M. , Kiuru, M. , Cairo, M. S. & Christiano, A. M. Generation of keratinocytes from normal and recessive dystrophic epidermolysis bullosa-induced pluripotent stem cells. Proc. Natl Acad. Sci. USA 108, 8797–8802 (2011). 21555586 10.1073/pnas.1100332108 PMC3102348

[bib19] Umegaki-Arao, N. et al. Induced pluripotent stem cells from human revertant keratinocytes for the treatment of epidermolysis bullosa. Sci. Transl. Med. 6, 264ra164 (2014). 10.1126/scitranslmed.3009342 25429057

[bib20] Kogut, I. , Roop, D. R. & Bilousova, G. Differentiation of human induced pluripotent stem cells into a keratinocyte lineage. Methods Mol. Biol. 1195, 1–12 (2014). 24510784 10.1007/7651_2013_64 PMC4096605

[bib21] Conget, P. et al. Replenishment of type VII collagen and re-epithelialization of chronically ulcerated skin after intradermal administration of allogeneic mesenchymal stromal cells in two patients with recessive dystrophic epidermolysis bullosa. Cytotherapy 12, 429–431 (2010). 20230217 10.3109/14653241003587637

[bib22] Sasaki, M. et al. Mesenchymal stem cells are recruited into wounded skin and contribute to wound repair by transdifferentiation into multiple skin cell type. J. Immunol. 180, 2581–2587 (2008). 18250469 10.4049/jimmunol.180.4.2581

[bib23] Schwarz, S. et al. Bone marrow-derived mesenchymal stem cells migrate to healthy and damaged salivary glands following stem cell infusion. Int. J. Oral Sci. 6, 154–161 (2014). 24810808 10.1038/ijos.2014.23 PMC4170149

[bib24] Wu, Y. , Chen, L. , Scott, P. G. & Tredget, E. E. Mesenchymal stem cells enhance wound healing through differentiation and angiogenesis. Stem Cells 25, 2648–2659 (2007). 17615264 10.1634/stemcells.2007-0226

[bib25] Fu, X. et al. Comparison of immunological characteristics of mesenchymal stem cells derived from human embryonic stem cells and bone marrow. Tissue Eng. Part A 21, 616–626 (2015). 25256849 10.1089/ten.tea.2013.0651 PMC4334098

[bib26] Jacobs, S. A. , Roobrouck, V. D. , Verfaillie, C. M. & Van Gool, S. W. Immunological characteristics of human mesenchymal stem cells and multipotent adult progenitor cells. Immunol. Cell Biol. 91, 32–39 (2013). 23295415 10.1038/icb.2012.64 PMC3540326

[bib27] Lian, Q. et al. Functional mesenchymal stem cells derived from human induced pluripotent stem cells attenuate limb ischemia in mice. Circulation 121, 1113–1123 (2010). 20176987 10.1161/CIRCULATIONAHA.109.898312

[bib28] Dominici, M. et al. Minimal criteria for defining multipotent mesenchymal stromal cells. The International Society for Cellular Therapy position statement. Cytotherapy 8, 315–317 (2006). 16923606 10.1080/14653240600855905

[bib29] Tolar, J. et al. Amelioration of epidermolysis bullosa by transfer of wild-type bone marrow cells. Blood 113, 1167–1174 (2009). 18955559 10.1182/blood-2008-06-161299 PMC2635082

[bib30] Fujita, Y. et al. Bone marrow transplantation restores epidermal basement membrane protein expression and rescues epidermolysis bullosa model mice. Proc. Natl Acad. Sci. USA 107, 14345–14350 (2010). 20660747 10.1073/pnas.1000044107 PMC2922560

[bib31] Genovese, P. et al. Targeted genome editing in human repopulating haematopoietic stem cells. Nature 510, 235–240 (2014). 24870228 10.1038/nature13420 PMC4082311

[bib32] Hoban, M. D. et al. Correction of the sickle cell disease mutation in human hematopoietic stem/progenitor cells. Blood 125, 2597–2604 (2015). 25733580 10.1182/blood-2014-12-615948 PMC4408287

[bib33] Wang, J. et al. Homology-driven genome editing in hematopoietic stem and progenitor cells using ZFN mRNA and AAV6 donors. Nat. Biotechnol. 33, 1256–1263 (2015). 26551060 10.1038/nbt.3408 PMC4842001

[bib34] Ditadi, A. et al. Human definitive haemogenic endothelium and arterial vascular endothelium represent distinct lineages. Nat. Cell Biol. 17, 580–591 (2015). 25915127 10.1038/ncb3161 PMC4551438

[bib35] Kennedy, M. et al. T lymphocyte potential marks the emergence of definitive hematopoietic progenitors in human pluripotent stem cell differentiation cultures. Cell Rep. 2, 1722–1735 (2012). 23219550 10.1016/j.celrep.2012.11.003

[bib36] Sturgeon, C. M. , Ditadi, A. , Awong, G. , Kennedy, M. & Keller, G. Wnt signaling controls the specification of definitive and primitive hematopoiesis from human pluripotent stem cells. Nat. Biotechnol. 32, 554–561 (2014). 24837661 10.1038/nbt.2915 PMC4152856

[bib37] Ng, E. S. , Davis, R. , Stanley, E. G. & Elefanty, A. G. A protocol describing the use of a recombinant protein-based, animal product-free medium (APEL) for human embryonic stem cell differentiation as spin embryoid bodies. Nat. Protoc. 3, 768–776 (2008). 18451785 10.1038/nprot.2008.42

[bib38] Osborn, M. et al. CRISPR/Cas9 Targeted Gene Editing and Cellular Engineering in Fanconi Anemia. Stem Cells Dev. 25, 1591–1603 (2016). 10.1089/scd.2016.0149 PMC5035838 27538887

[bib39] Schmitt, T. M. et al. Induction of T cell development and establishment of T cell competence from embryonic stem cells differentiated *in vitro*. Nat. Immunol. 5, 410–417 (2004). 15034575 10.1038/ni1055

[bib40] Gori, J. L. et al. Vascular niche promotes hematopoietic multipotent progenitor formation from pluripotent stem cells. J. Clin. Invest. 125, 1243–1254 (2015). 25664855 10.1172/JCI79328 PMC4362238

[bib41] Sandler, V. M. et al. Reprogramming human endothelial cells to haematopoietic cells requires vascular induction. Nature 511, 312–318 (2014). 25030167 10.1038/nature13547 PMC4159670

[bib42] Kuhl, T. et al. High local concentrations of intradermal MSCs restore skin integrity and facilitate wound healing in dystrophic epidermolysis bullosa. Mol. Ther. 23, 1368–1379 (2015). 25858020 10.1038/mt.2015.58 PMC4817872

[bib43] Tolar, J. et al. Induced pluripotent stem cells from individuals with recessive dystrophic epidermolysis bullosa. J. Invest. Dermatol. 131, 848–856 (2011). 21124339 10.1038/jid.2010.346 PMC4151825

[bib44] Osborn, M. J. et al. TALEN-based gene correction for epidermolysis bullosa. Mol. Ther. 21, 1151–1159 (2013). 23546300 10.1038/mt.2013.56 PMC3677309

[bib45] Izmiryan, A. , Danos, O. & Hovnanian, A. Meganuclease-mediated *COL7A1* gene correction for recessive dystrophic epidermolysis bullosa. J. Invest. Dermatol. 136, 872–875 (2016). 26897595 10.1016/j.jid.2015.11.028

[bib46] Sebastiano, V. et al. Human *COL7A1*-corrected induced pluripotent stem cells for the treatment of recessive dystrophic epidermolysis bullosa. Sci. Transl. Med. 6, 264ra163 (2014). 10.1126/scitranslmed.3009540 PMC4428910 25429056

[bib47] Chamorro, C. et al. Gene editing for the efficient correction of a recurrent *COL7A1* mutation in recessive dystrophic epidermolysis bullosa keratinocytes. Mol. Ther. Nucleic Acids 5, e307 (2016). 27045209 10.1038/mtna.2016.19 PMC5014520

[bib48] Wenzel, D. et al. Genetically corrected iPSCs as cell therapy for recessive dystrophic epidermolysis bullosa. Sci. Transl. Med. 6, 264ra165 (2014). 10.1126/scitranslmed.3010083 25429058

[bib49] Goto, M. et al. Fibroblasts show more potential as target cells than keratinocytes in COL7A1 gene therapy of dystrophic epidermolysis bullosa. J. Invest. Dermatol. 126, 766–772 (2006). 16439972 10.1038/sj.jid.5700117

[bib50] Lin, S. , Staahl, B. T. , Alla, R. K. & Doudna, J. A. Enhanced homology-directed human genome engineering by controlled timing of CRISPR/Cas9 delivery. Elife 3, e04766 (2014). 25497837 10.7554/eLife.04766 PMC4383097

[bib51] Heyer, W. D. , Ehmsen, K. T. & Liu, J. Regulation of homologous recombination in eukaryotes. Annu. Rev. Genet. 44, 113–139 (2010). 20690856 10.1146/annurev-genet-051710-150955 PMC4114321

[bib52] van Rensburg, R. et al. Chromatin structure of two genomic sites for targeted transgene integration in induced pluripotent stem cells and hematopoietic stem cells. Gene Ther. 20, 201–214 (2013). 22436965 10.1038/gt.2012.25 PMC3661409

[bib53] Fujie, Y. et al. New type of sendai virus vector provides transgene-free iPS cells derived from chimpanzee blood. PLoS ONE 9, e113052 (2014). 25479600 10.1371/journal.pone.0113052 PMC4257541

[bib54] Mali, P. et al. RNA-guided human genome engineering via Cas9. Science 339, 823–826 (2013). 23287722 10.1126/science.1232033 PMC3712628

[bib55] Gibson, D. G. et al. Enzymatic assembly of DNA molecules up to several hundred kilobases. Nat. Methods 6, 343–345 (2009). 19363495 10.1038/nmeth.1318

[bib56] Guschin, D. Y. et al. A rapid and general assay for monitoring endogenous gene modification. Methods Mol. Biol. 649, 247–256 (2010). 20680839 10.1007/978-1-60761-753-2_15

[bib57] Tolar, J. et al. Keratinocytes from induced pluripotent stem cells in junctional epidermolysis bullosa. J. Invest. Dermatol. 133, 562–565 (2013). 22931927 10.1038/jid.2012.278 PMC3514565

