# Supplementary material for: CRISPR/Cas9-based genetic correction for recessive dystrophic epidermolysis bullosa
Source: NPJ Regen Med. 2016 Dec 8;1:16014–. doi: 10.1038/npjregenmed.2016.14 (PMC5328670; doi:10.1038/npjregenmed.2016.14)
Supplement: Supplementary Figures [file npjregenmed201614-s1.pdf]

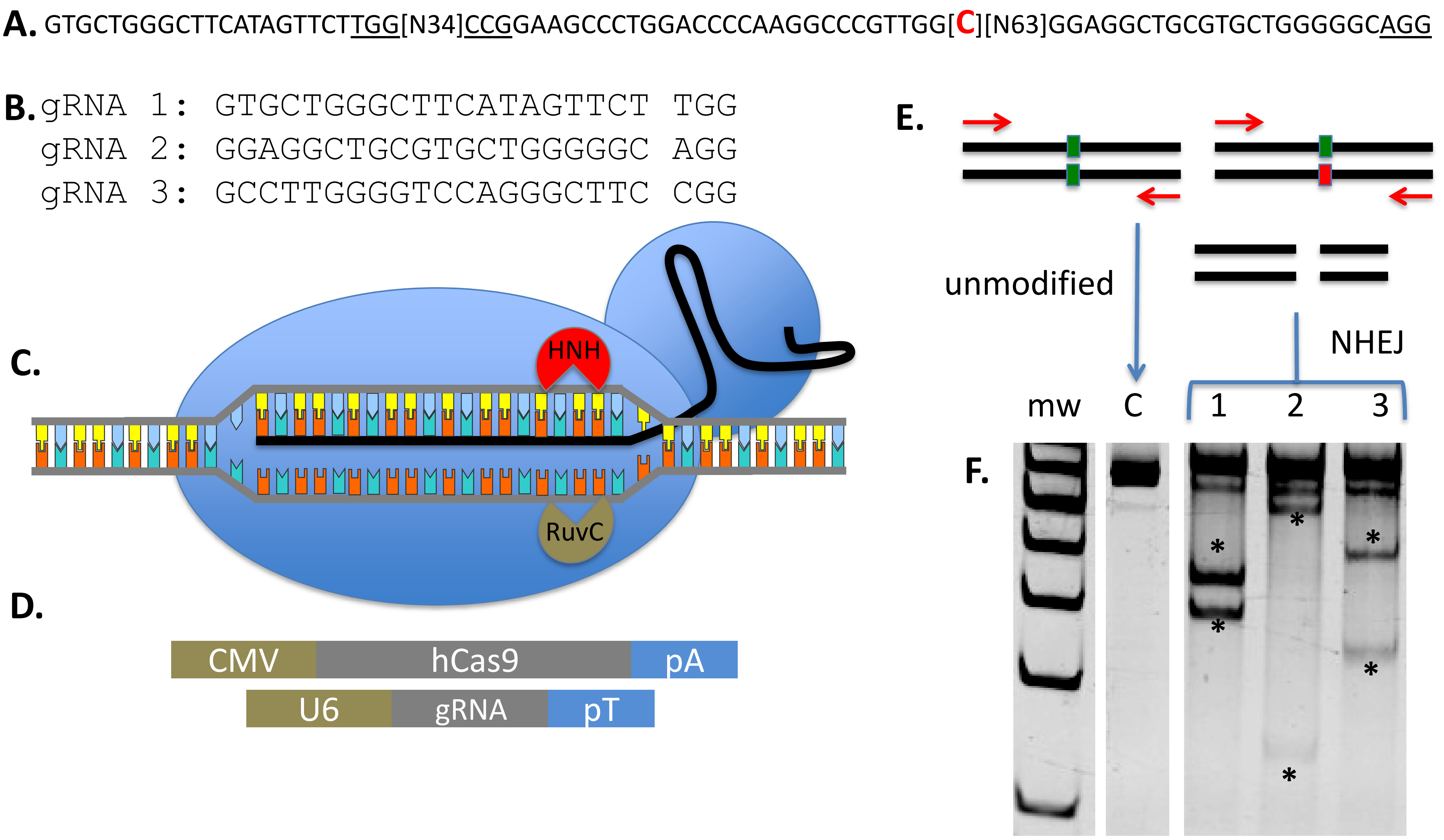

Figure S1. CRISPR/Cas9 candidate testing. (A) *COL7A1* gene sequence and CRISPR/Cas9 target sites. The cytosine that is deleted in the RDEB-causing 4317elC genotype is shown in red. The protospacer adjacent motifs (PAM) for the CRISPR/Cas9 guide RNAs are underlined and proximity to the mutation is shown numerically in brackets. (B) CRISPR/Cas9 candidates. The three gene targets with a space between the PAM sequences are shown. (C) CRISPR/Cas9 architecture. The gRNA (black line) recognizes a target site via Watson-Crick base pairing and complexes with the Cas9 nuclease protein (blue circles) that contain two domains (HNH and RuvC) that cleave a single strand of DNA. Inactivation of the HNH domain (indicated by red coloring) by a D10A substitution allows for a 'nicking' version of Cas9 that cuts one strand of DNA. (D) CRISPR/Cas9 gene delivery. The Cas9 nuclease or nickase were delivered on a plasmid under the control of the CMV promoter and bovine growth hormone polyadenylation signal (pA). The gRNA was expressed on a second plasmid from the U6 polIII promoter and a polyT transcriptional terminator. (E) Surveyor nuclease assay. CRISPR/Cas9 treated 293T cells were analyzed using Surveyor methodology by amplifying the *COL7A1* region for which the gRNAs were designed. Modified (red box) and unmodified (green box) in the amplicon pool were annealed and NHEJ-mediated indels led to imperfect pairing and cleavage of the DNA by Surveyor enzyme. (F) Polyacrylamide gel of Surveyor assay in (E).

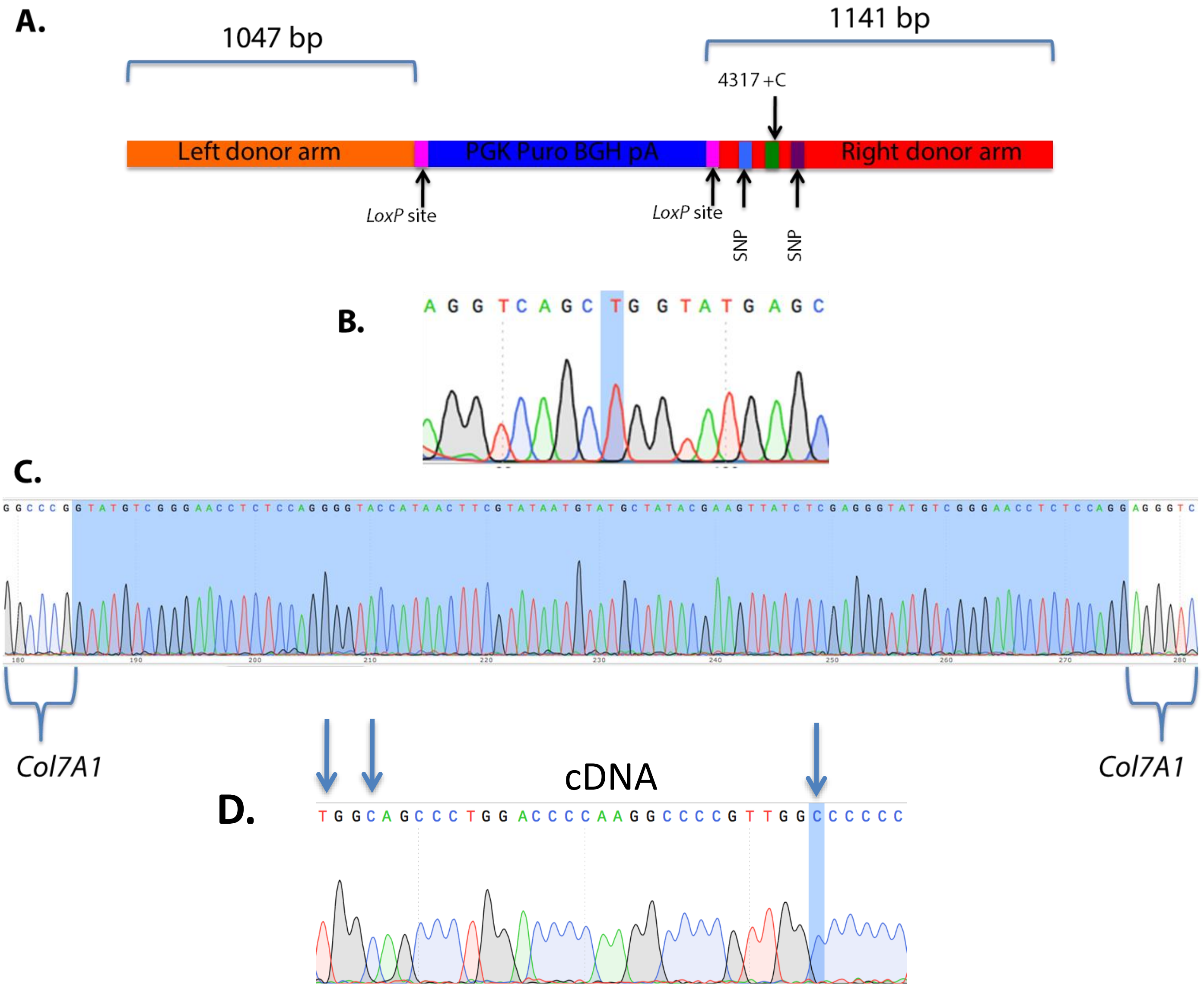

Figure S2. HDR donor and analysis. (A) Plasmid donor. Left (orange box, 1047 bp in length) and right arms (1141 bp in length, red box) of homology flanked an autonomous floxed (pink boxes) murine phosphoglycerate kinase 1 promoter driven puromycin resistance gene with a bovine growth hormone polyadenylation signal (blue box). The right donor arm contained two silent polymorphisms (SNP) and a sequence to restore the proper wild-type gene sequence at the 4317 delC locus. (B) Inside-out PCR sequencing. PCR primers to document HDR were employed with one inside the donor in the PGK sequence and the second outside the right donor arm. The blue highlighting shows the demarcation between donor sequence and endogenous *COL7A1* locus sequence. (C) Cre excision sequence. iPSCs subjected to cre mRNA electroporation were analyzed for the cre footprint (shaded region) contained to the intron. Included also were non-human CRISPR/Cas9 sequence as marker sequences. The endogenous COL7A1 sequences are shown with brackets at the termini. (D) cDNA analysis. RNA from gene-corrected cells was analyzed for proper sequence documentation. Corrected base is highlighted in blue, and SNPs are indicated with arrows.

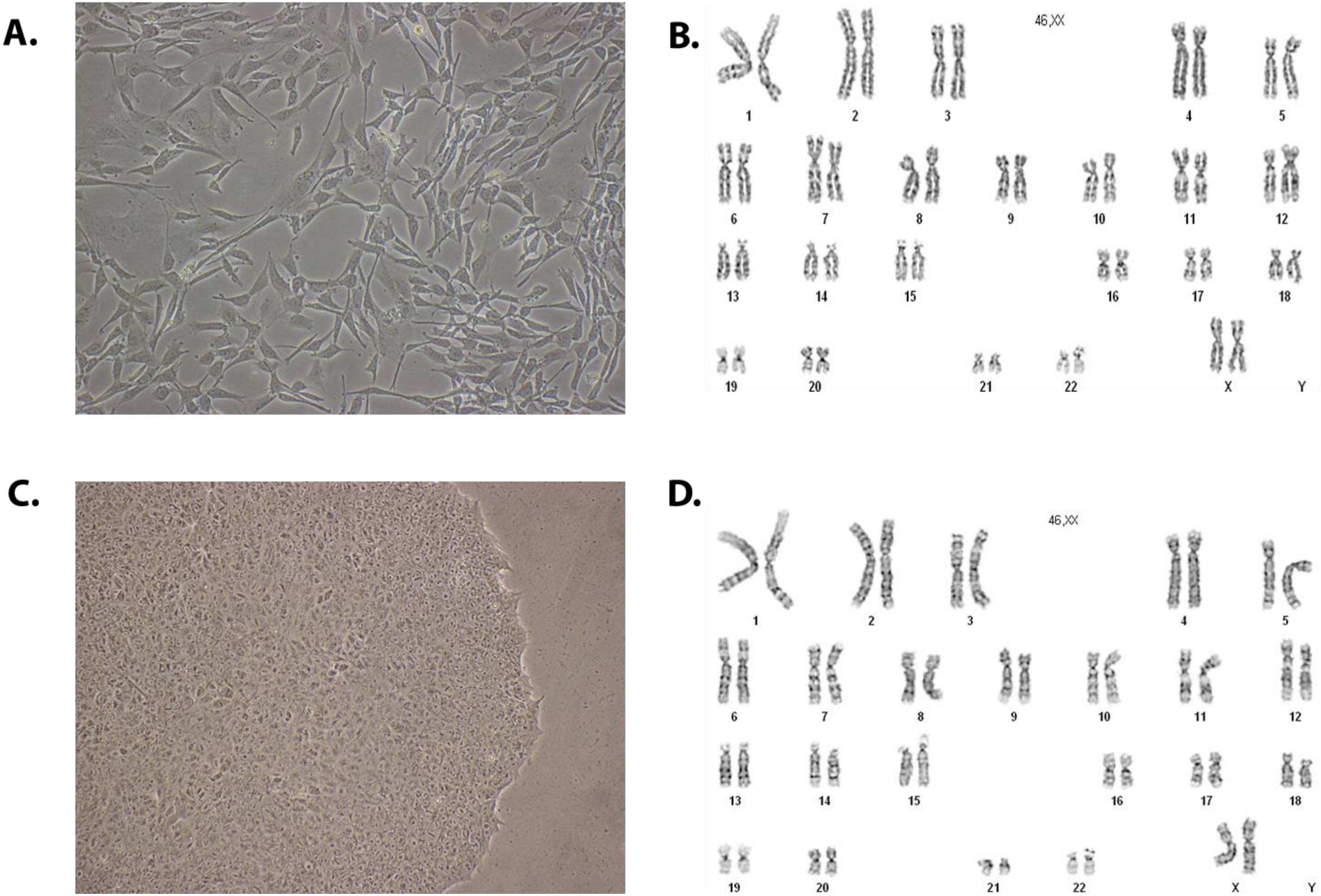

Figure S3. Morphology and karyotype of patient-specific cells. (A) Gene-corrected fibroblasts are shown with a correct phenotype (B). (C) iPSC colony morphology with defined and rounded borders with (D) a 46XX karyotype.

Lane 1: mw stds
Lane 2: Nuclease
Lane 3: Nickase
Lane 4: GFP Ctrl

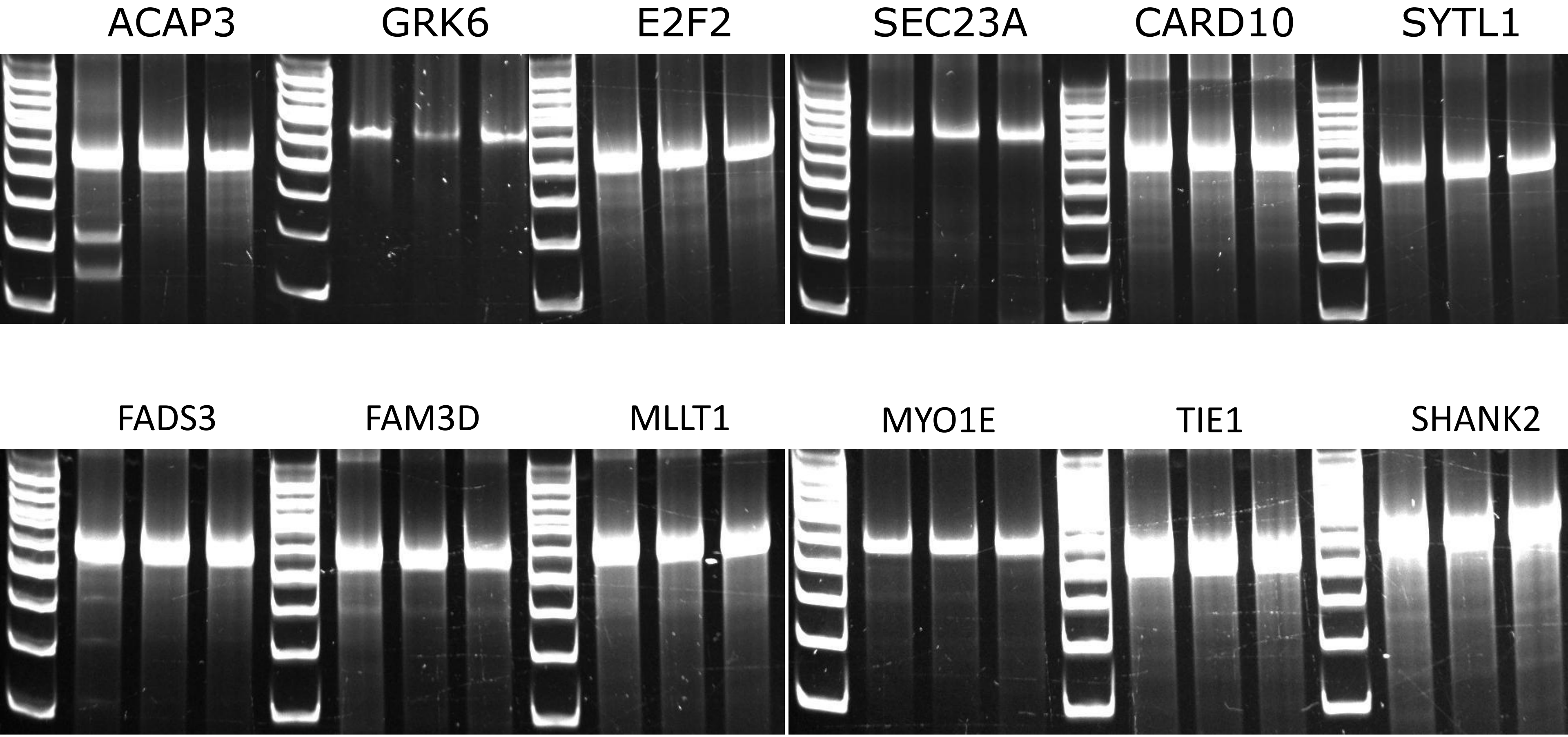

Figure S4. Off-target analysis. HEK 293T cells were treated with the *COL7A1* gRNA and the Cas9 nuclease, nickase, or a GFP transfection control plasmid. At 72 hours the predicted off-target loci were amplified by PCR and analyzed by the Surveyor method. Representative gel images with identical exposure rates are shown.

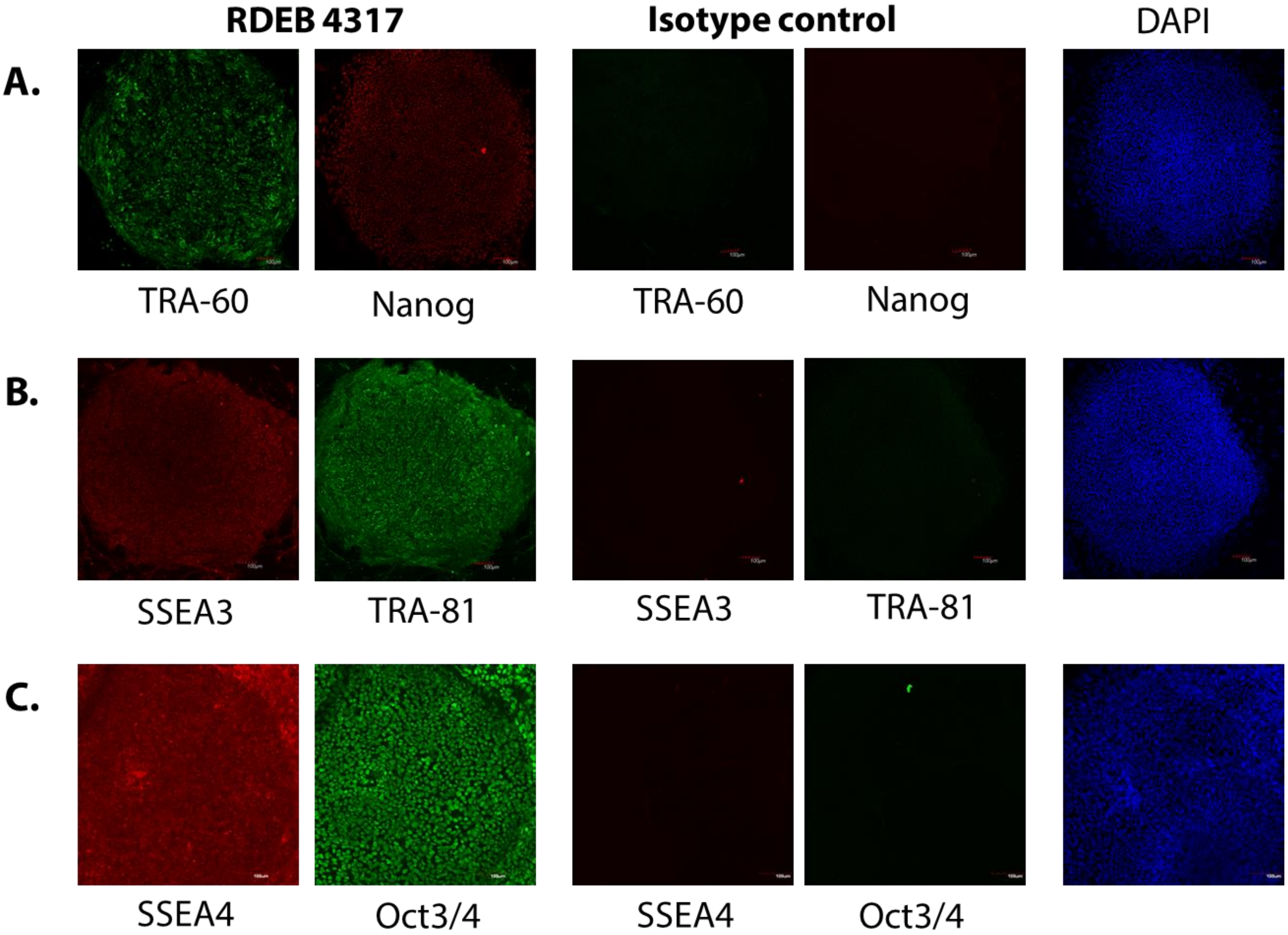

Figure S5. iPSC phenotype. iPSCs were analyzed for pluripotent markers by immunofluorescence.

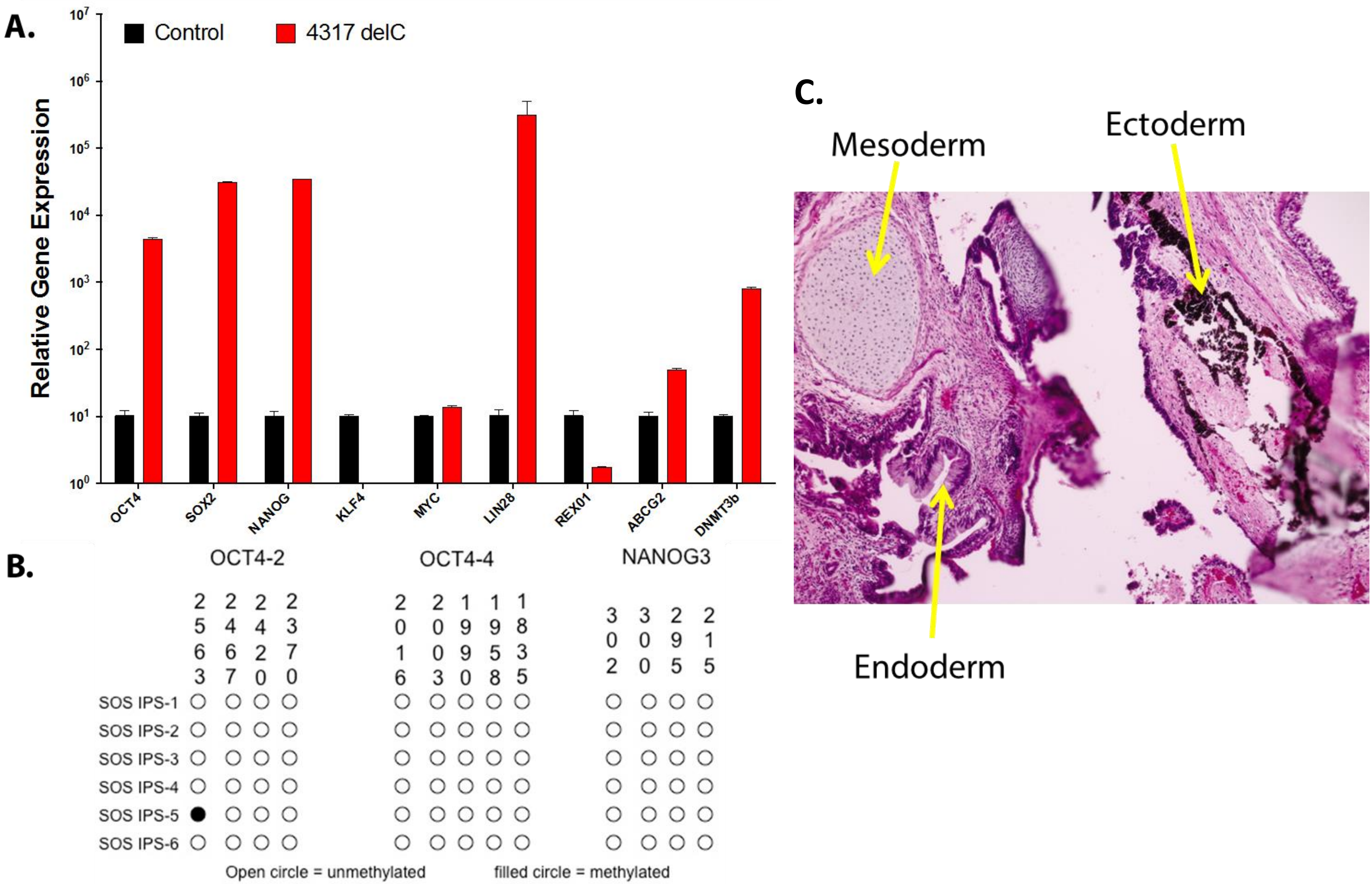

Figure S6. iPSC gene and epigenome analysis. (A) TaqMan gene expression. qRT-PCR analysis of reprogramming associated genes was performed (n=3 replicates) in patient-derived fibroblasts and iPSCs. (B) Promoter methylation status. Sodium bisulfite treated DNA from patient-derived iPSCs (analyzed in triplicate with a representative image shown) was analyzed for the presence of methylated cytosines at the CpG islands in the *OCT4* and *NANOG* gene promoters. (C) iPSC-derived teratoma. iPSCs were injected into the flank of NOD/scid IL2Rg null mice and excised. Shown is a representative image from 3-5 mice.

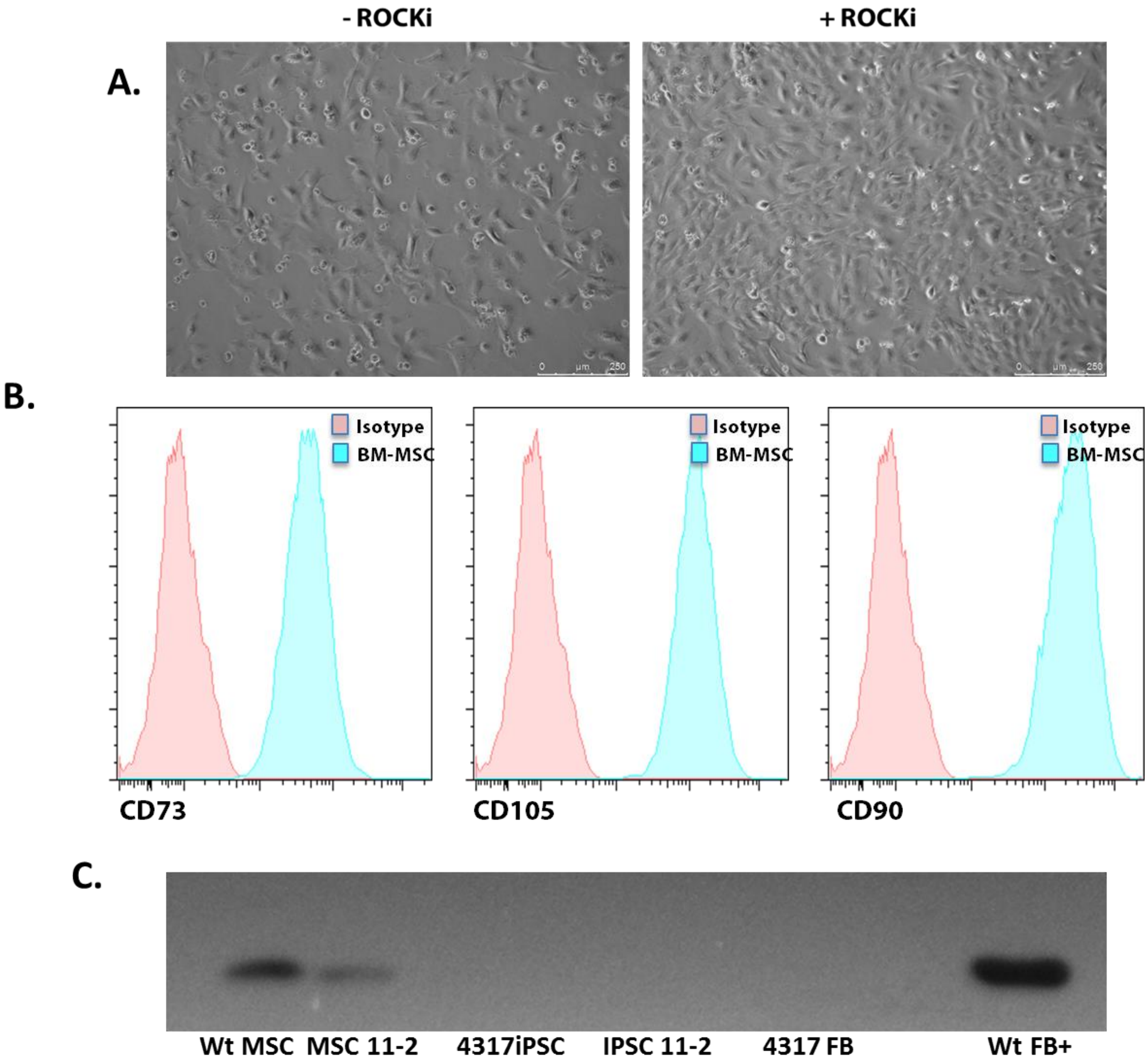

Figure S7. MSC analysis. (A) ROCK inhibition enhances re-plating and survival of iPSC-MSCs. Images of iPSC-MSCs after the first passage with and without ROCK inhibition. (B) Bone marrow MSCs. MSCs were derived from a bone marrow aspirate from a human donor and assessed for cell surface expression of CD73, CD105, and CD90.

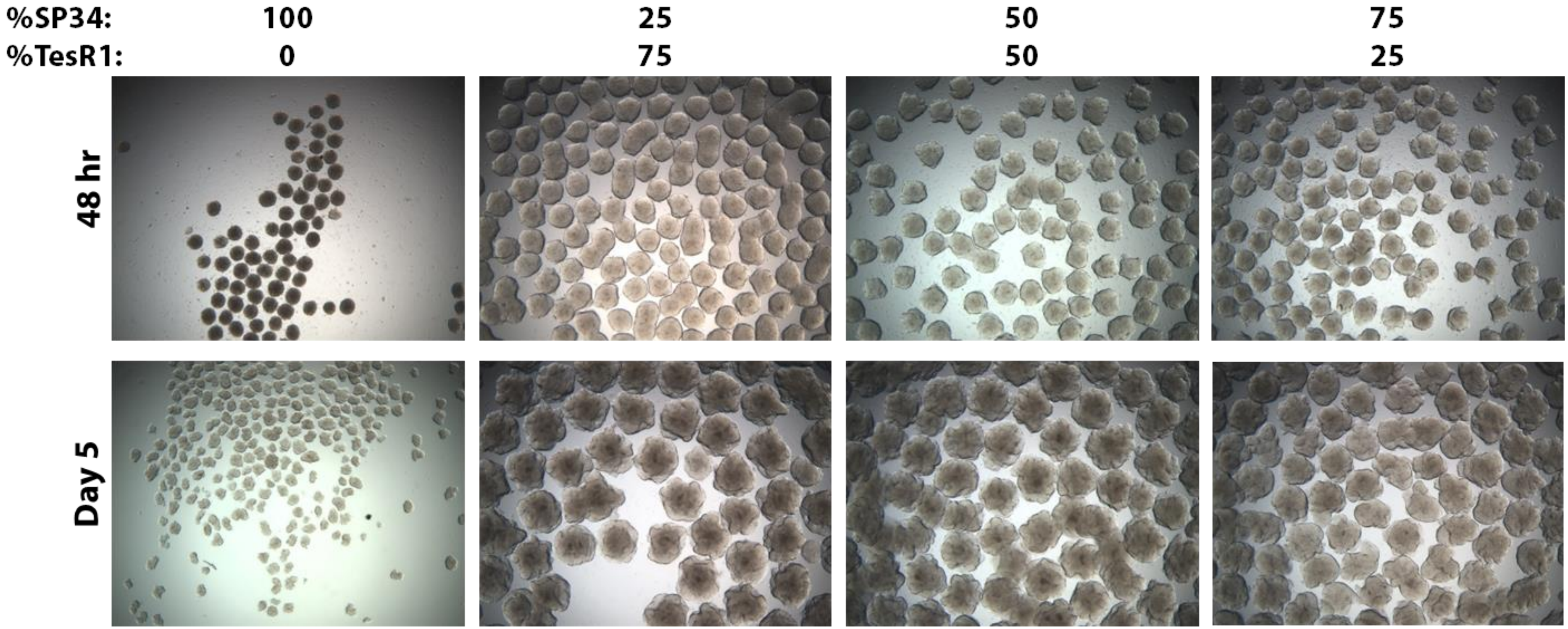

Figure S8: Inclusion of Tesr1 enhances EB formation and growth. Embryoid bodies were formed in StemPro34 media supplemented with indicated amounts of Tesr1 and imaged at indicated timepoints. Images are at 50x magnification.

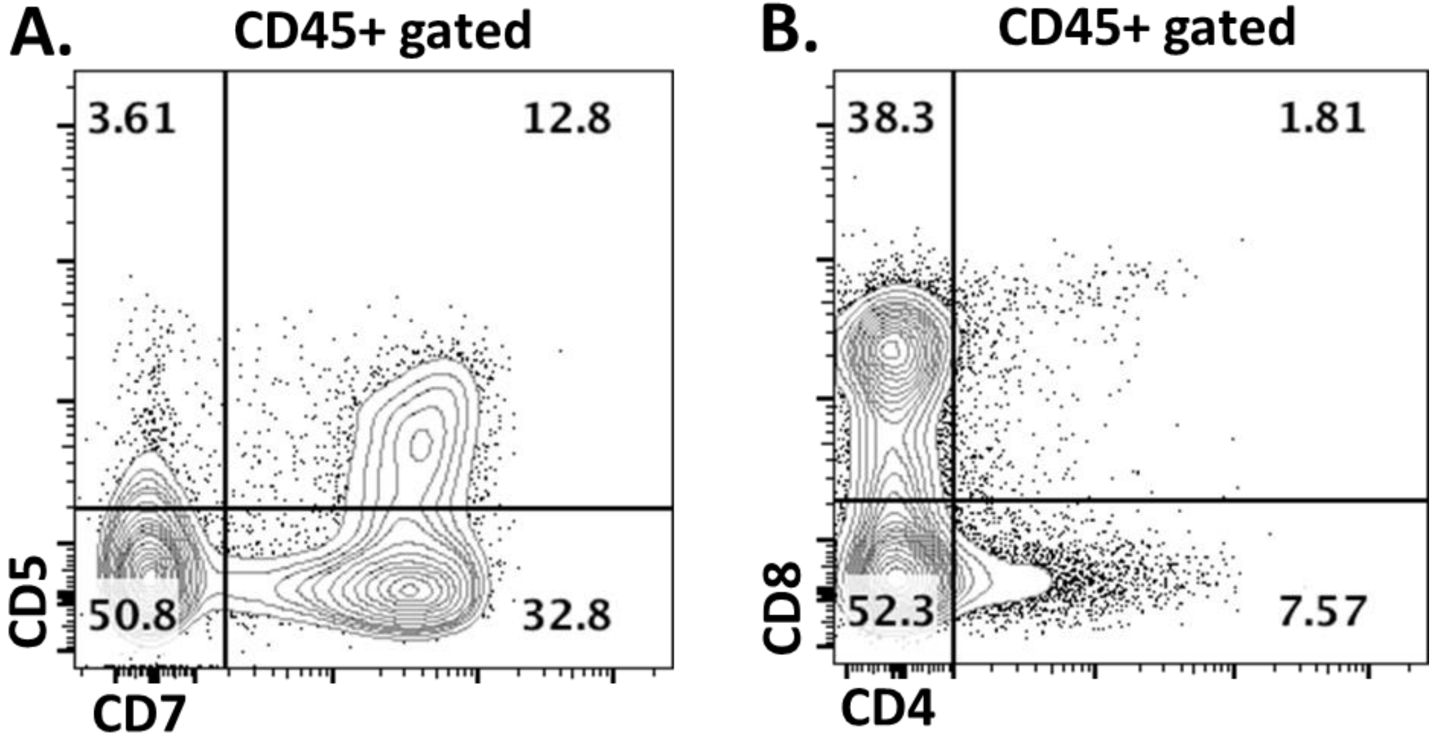

Figure S9. T-lineage differentiation. (A) Embryoid body-derived CD34+ cells were co-cultured with OP9-DLL4 and analyzed for early T-lineage specification by CD7 and CD5 expression. (B) Formation of CD4 and CD8+ fractions after extended OP9-DLL4 co-culture.
